# Large-scale transcriptome comparison reveals distinct gene activations in wheat responding to stripe rust and powdery mildew

**DOI:** 10.1186/1471-2164-15-898

**Published:** 2014-10-15

**Authors:** Hong Zhang, Yongzheng Yang, Changyou Wang, Min Liu, Hao Li, Ying Fu, Yajuan Wang, Yingbin Nie, Xinlun Liu, Wanquan Ji

**Affiliations:** State Key Laboratory of Crop Stress Biology for Arid Areas, College of Agronomy (Northwest A&F University), Yangling, Shaanxi 712100 China

**Keywords:** Bread wheat, Stripe rust, Powdery mildew, RNA-Seq, Gene expression

## Abstract

**Background:**

Stripe rust (*Puccinia striiformis* f. sp. *tritici*; *Pst*) and powdery mildew (*Blumeria graminis* f. sp. *tritici*; *Bgt*) are important diseases of wheat (*Triticum aestivum*) worldwide. Similar mechanisms and gene transcripts are assumed to be involved in the host defense response because both pathogens are biotrophic fungi. The main objective of our study was to identify co-regulated mRNAs that show a change in expression pattern after inoculation with *Pst* or *Bgt*, and to identify mRNAs specific to the fungal stress response.

**Results:**

The transcriptome of the hexaploid wheat line N9134 inoculated with the Chinese *Pst* race CYR 31 was compared with that of the same line inoculated with *Bgt* race E09 at 1, 2, and 3 days post-inoculation. Infection by *Pst* and *Bgt* affected transcription of 23.8% of all *T. aestivum* genes. Infection by *Bgt* triggered a more robust alteration in gene expression in N9134 compared with the response to *Pst* infection. An array of overlapping gene clusters with distinctive expression patterns provided insight into the regulatory differences in the responses to *Bgt* and *Pst* infection. The differentially expressed genes were grouped into seven enriched Kyoto Encyclopedia of Genes and Genomes pathways in *Bgt*-infected leaves and four pathways in *Pst*-infected leaves, while only two pathways overlapped. In the plant–pathogen interaction pathway, N9134 activated a higher number of genes and pathways in response to *Bgt* infection than in response to *Pst* invasion. Genomic analysis revealed that the wheat genome shared some microbial genetic fragments, which were specifically induced in response to *Bgt* and *Pst* infection.

**Conclusions:**

Taken together, our findings indicate that the responses of wheat N9134 to infection by *Bgt* and *Pst* shows differences in the pathways and genes activated. The mass sequence data for wheat–fungus interaction generated in this study provides a powerful platform for future functional and molecular research on wheat–fungus interactions.

**Electronic supplementary material:**

The online version of this article (doi:10.1186/1471-2164-15-898) contains supplementary material, which is available to authorized users.

## Background

Plants are constantly exposed to microbes in natural and agricultural ecosystems. To be pathogenic, most microbes must access the plant’s internal tissues, either by penetrating the plant surface directly or by entering through wounds or natural openings such as stomata [1, 2]. Furthermore, the pathogen must pierce through the cell wall, and often the host plasma membrane, to be infective. Plants wield typical basal and resistance (R) gene-mediated defense mechanisms and mount a defensive response to pathogen attack to delay or arrest potential pathogenic microorganism growth [3] through pathogen-associated molecular pattern-triggered immunity (PTI) and effector-triggered immunity (ETI) systems. Current analysis of plant immunity has moved towards an integrated view of plant–pathogen interactions [4]. Using oligonucleotides, cDNA microarrays and transcriptome analysis, many comprehensive analyses of stress-induced changes in gene expression in model plants with small genomes have been undertaken, and consequently many genes associated with pathogenic stress responses in plants are known. However, numerous fundamental molecular aspects remain unknown, such as the unique and common signaling components of PTI and ETI, and the induced host components that effect resistance. A global gene expression approach is useful for elucidating the molecular mechanisms of wheat–fungus interactions, particularly the application of next-generation sequencing to study important non-model host–pathogen systems, such as wheat rusts [5].

Stripe rust (*Puccinia striiformis* f. sp. *tritici*; *Pst*) and powdery mildew (*Blumeria graminis* f. sp. *tritici*; *Bgt*) are important fungal diseases of wheat (*Triticum aestivum*) in many wheat-growing regions of the world. The filamentous powdery mildew and rust fungi develop haustoria within the lumen of the host cell, which function to absorb nutrients. A number of studies on wheat–rust interactions have been carried out using the Affymetrix® GeneChip® Wheat Genome Array (Affymetrix, Santa Clara, CA, USA) [6, 7] and cDNA-AFLP analysis [8]. Powdery mildew infection has been studied in grapevine [9], *Hordeum* [10] and *Arabidopsis* [11] with the Affymetrix GeneChip Wheat Genome Array, cDNA-AFLP, and cDNA microarrays, respectively. However, a complete gene expression profile for the response to the stripe rust and powdery mildew pathogens in the same wheat germplasm is still lacking. Compared with other plants with smaller genomes, the use of most genetic and molecular techniques to study genes involved in wheat–*Pst* or wheat–*Bgt* interactions has been limited, because hexaploid wheat has a large and complex genome and its transformation is difficult, and both fungi show sexual reproduction and irreversible deletion of genes dispensable for biotrophy [10, 12]. Use of the Affymetrix GeneChip Wheat Genome Array is often restricted by the known gene sequences arrayed on the chip, with a limited number of expressed sequence tags (ESTs) non-specific to different wheat materials, whereas cDNA-AFLP is universally applicable for any organism or interaction without prior sequence information required, although false positives may frequently be observed because of technical reasons. In contrast, RNA sequencing (RNA-Seq) is not dependent on pre-existing databases of expressed genes and, therefore, provides an unbiased view of gene expression profiles. In the present study, using Illumina deep sequencing, we undertook a transcriptome analysis of leaves from different plants of the same wheat line, N9134, subjected to both *Pst* and *Bgt* stress treatments. The main objective was to identify co-regulated mRNAs that show a change in expression pattern after inoculation with *Pst* or *Bgt*, and to identify mRNAs specific to the fungal stress response. This is the first study to compare two biotic stresses using a global expression profiling strategy in the same wheat genetic background. Through a comparison of different pathogen stress treatments with biological replicates, we reasoned that we could better identify both shared and stimulus-specific responses.

## Results

In the present study, cDNA libraries were constructed from leaves inoculated with *Pst* or *Bgt* at 0, 1, 2 and 3 days post-inoculation (dpi) with three biological replicates, and then sequenced using the Illumina HiSeq™ 2000 platform. After cleaning and checking the read quality, we obtained almost 46.75 million 101 bp paired-end clean reads. Among the clean reads, 100% had quality scores at the Cycle Q20 level (a base quality greater than 20 and an error probability of 0.01). The data sets were deposited in the NCBI Sequence Read Archive (accession number PRJNA243835). Because of deficiencies in the reference genome sequence, these reads were *de novo* assembled using the Trinity platform software, resulting in 186,632 unigenes with N50 length of 743 bp, of which 89,672 unigenes were annotated after Blast searches of the GenBank Nr, SwissProt, KEGG, COG and GO databases. The length of 22,825 unigenes was more than 1 kb and contained 4,837 simple sequence repeat sites.

As an aid to examining gene expression level distributions, the reads per kilobase of exon model per million of aligned reads (RPKM) values were calculated as normalized expression estimates for each gene model in each sample. Also, correlation coefficients were calculated based on the log-transformed RPKM values after eliminating genes with a zero count in any of the three replicates. The correlation coefficient values ranged from 0.930 to 0.994 (Additional file 1: Table S1), indicating there was a strong correlation between replicates. A generalized linear model was applied based on a negative binomial distribution and an overall test was conducted to determine which genes varied in expression among any of the seven treatment groups, where a treatment group was defined by a strain-by-induction condition combination (see Methods for details). Setting fold change ≥2 and the false discovery rate (FDR) at 1.0% using the method of Benjamini and Hochberg [13], statistical analysis with DESeq identified 10,583 genes as differentially expressed among the six treatment groups compared with non-inoculated leaves as the control. Of these genes, the length of 7,298 genes exceeded 1 kb. Table 1 lists details of the differentially expressed genes (DEGs) and annotation numbers that were detected at the different time points in response to the fungal stress treatments.

**Table 1:** **Statistical table of differentially expressed genes number and annotated DEGs**

Type	S1_vs_Ck	S2_vs_Ck	S3_vs_Ck	P1_vs_Ck	P2_vs_Ck	P3_vs_Ck
**num**	2,208	991	1,065	4,549	5,275	5,525
**up**	900	324	326	2,771	1,548	1,718
**down**	1,308	667	739	1,778	3,727	3,807
**nr**	2,088	913	1,011	4,213	4,789	4,882
**SwissProt**	1,811	765	869	3,700	4,058	4,029
**GO**	1,871	783	894	3,786	4,181	4,213
**KEGG**	432	168	228	888	868	899
**COG**	853	301	375	1,685	1,696	1,660

Note: P represents powdery mildew E09 inoculation condition; S represents stripe rust pathogen CYR 31 inoculation; Ck represents the samples without pathogen stress as control. The number of 1, 2 and 3: N9134 infected at 1, 2 and 3 dpi, respectively.

To evaluate the reliability of our RNA-Seq and *de novo* assembled results, quantitative real-time PCR (qPCR) was performed on eight selected genes of interest using RNA samples as a fourth replication. These genes were selected to represent a wide range of expression levels and patterns under fungal infection. Six gene expression patterns in response to *Bgt* stress showed strong agreement and were highly correlated in the RNA-Seq and qPCR analyses (Additional file 1: Figure S1). Additionally, six previously studied full-length genes were aligned with the present assembled unigene database and showed identities of up to 99.6%.

### More robust response to *Bgt*- than *Pst*-induced stress in bread wheat

**Figure 1:**
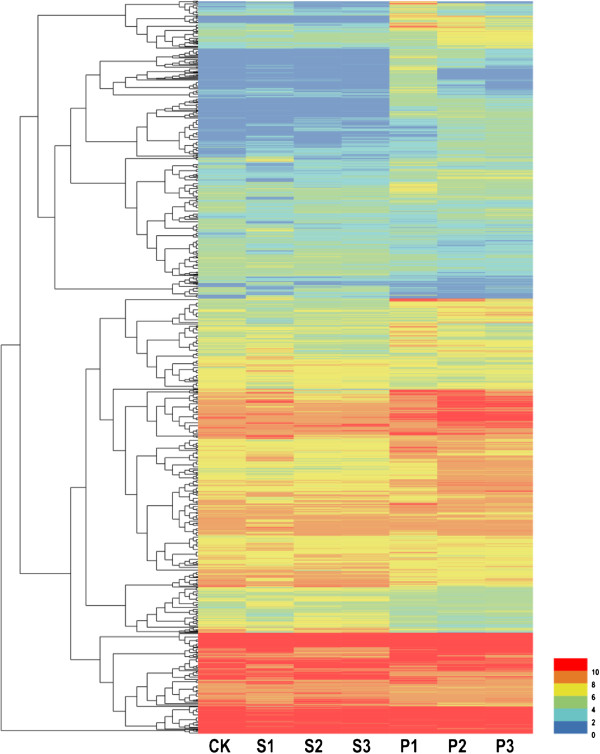
**Hierarchical clustering of differentially-expressed genes (DEGs) in resistant bread wheat accession N9134 to** ***Bgt*** **and** ***Pst*** **infection.** The signal ratios are shown in a red-green color scale, where red represents up-regulation and green represents down-regulation. Each column (Ck, S1, S2, S3, P1, P2 and P3) represents the mean expression value of the RNA-Seq obtained from three biological replicates and each row represents a differentially-expressed genes. Ck: resistant wheat N9134 exempt from pathogen stress as control; S1, S2 and S3: N9134 infected with stripe rust pathogen CYR 31 at 1, 2 and 3 dpi, respectively; P1, P2 and P3: N9134 infected with powdery mildew pathogen E09 at 1, 2 and 3 dpi, respectively.

Although transcript levels do not necessarily reflect the amount of final active protein product, for simplicity, increased transcript abundance is often referred to as induced gene expression. Genes that were differentially expressed between non-inoculated and inoculated leaves were determined with RNA-Seq. Cluster analysis showed that the expression profiles of DEGs varied significantly in response to *Bgt* and *Pst* in the N9134 genotype (Figure 1).

For the *Bgt* test, using inoculated leaf samples, expression of 9,114 DEGs was detected, of which 6,292 genes exceeded 1 kb. As shown in Table 1 and (Additional file 1: Figure S2), comparison of inoculated and non-inoculated leaves showed that 4,549, 5,275 and 5,525 genes were differentially expressed at 1, 2, and 3 dpi, respectively. Of these genes, 2,771 and 1,778 were up- and down-regulated at 1 dpi, 1,548 and 3,727 at 2 dpi, and 1,718 and 3,807 at 3 dpi, respectively. In the *Pst* CYR 31 inoculation treatment, 3,359 DEGs showed significantly lower expression than that in the *Bgt* E09 stress treatment. Of these genes, 2,208 (900 up- and 1,308 down-regulated), 991 (324 up- and 667 down-regulated), and 1,065 (326 up- and 739 down-regulated) were differentially expressed at 1, 2, and 3 dpi, respectively. The number of up-regulated genes at 1 dpi was almost double those at 2 and 3 dpi after infection by *Bgt*, whereas the number at 1 dpi was almost three-times higher than those at 2 and 3 dpi after *Pst* infection. This substantiated the contention that 1 dpi is the most important time point for wheat to respond to fungal attack by expression of race-specific resistance genes.

To further test the variation in response to *Pst* and *Bgt* inoculation, we compared gene expression in response to *Pst* CYR 31 versus *Bgt* E09 inoculation with fold change ≥4 and FDR at 1.0%. Additional file 1: Figure S3 shows that 8,110 genes were differentially expressed in response to *Bgt* infection in comparison with *Pst* infection, of which 2,208 and 804 genes were up- and down-regulated at 1 dpi, 1,855 and 2,456 at 2 dpi, and 1,725 and 1,973 at 3 dpi, respectively. This result indicated that the wheat resistance response to *Bgt* inoculation triggered more robust alteration in gene expression than that observed in response to *Pst* inoculation.

### Gene characterization of stimulus-specific responses to *Pst*or *Bgt*

To facilitate a closer comparison of *Pst*-induced genes with *Bgt*-induced genes, a pairwise comparison was conducted after DEGs were annotated using KEGG classifications and GO assignments. All resistance-specific genes were analyzed using MAS to identify the metabolic pathways in which they function. The GO enrichment results are shown in Additional file 1: Tables S2 (biological process), S3 (molecular function), and S4 (cellular component). When the FDR-corrected *P*-value was set at 0.05, seven significant enriched KEGG pathways for *Bgt*-induced stress and four for *Pst*-induced stress versus the non-stressed control were identified. Two KEGG pathways overlapped between the two infection treatments (Table 2), namely ubiquinone and other terpenoid-quinone biosynthesis and photosynthesis-antenna proteins. As there were insufficient pathway annotations in KEGG, the pathways were retrieved using the DEGs comparing *Pst* infection versus *Bgt* infection directly at the specific time points and are listed in Additional file 1: Table S5. Taken together, 16 KEGG pathways were identified.

**Table 2:** **Significant KEGG pathway of stimulus-specific responses on** ***Pst*** **or** ***Bgt***

KEGG pathway	Correct-p value					No of dysregulated enzymes
	P	P only	PS	S only	S	
ko00360 Phenylalanine metabolism	0.00055*	0.00691*	1	1(3.06)	1	10
ko00940 Phenylpropanoid biosynthesis	0.00559*	0.02167*	1	1	1	30
ko00592 alpha-Linolenic acid metabolism	0.01487*	0.00516*	1	1	1	13
ko00130 Ubiquinone and other terpenoid-quinone biosynthesis	0.01643*	1	0.01345*	1	0.10249	7
ko00196 Photosynthesis - antenna proteins	0.00570*	1	1.89E-05*	1	0.00138*	12
ko00941 Flavonoid biosynthesis	0.03179*	0.10650	1	1	1	25
Ko00400 Phenylalanine, tyrosine and tryptophan biosynthesis	0.06556	0.03537*	1	1	1	19
Ko00710 Carbon fixation in photosynthetic organisms	1	1	6.24E-05*	1	0.00296*	22
Ko00906 Carotenoid biosynthesis	1	1	1	0.72054	0.03038*	20
ko00910 Nitrogen metabolism	0.16142	1	1	-	1	14
ko00904 Diterpenoid biosynthesis	0.25882	0.12611	1	-	1	17
ko00945 Stilbenoid, diarylheptanoid and gingerol biosynthesis	0.27716	0.0556	1	-	1(2.5)	7
Ko03010 Ribosome	1	1	1(4.43)	0.92345	1	70
ko00280 Valine, leucine and isoleucine degradation	1	1	0.761726	1	1	27
ko00195 Photosynthesis	1	1	0.465078	-	1	24
ko00402 Benzoxazinoid biosynthesis	1	1	1	-	1	2

Note: All KEGG pathways were retrieved from MAS3.0 molecule annotation system. Some plant-specific pathways were computed repeat by extracting genes from comparing *Pst* with *Bgt* stress. The values of Correct-p were given and those less than 0.05 were marked with asterisks. Some enriched KEGG pathways with the high enrichment factor were noted and the factor was given in brackets. The indistinctive pathway that was listed (bottom 7 lines) had p-value <0.05 for analysis DEGs from *Pst* vs *Bgt*.

Phenylalanine metabolism, phenylpropanoid biosynthesis, alpha-linolenic acid metabolism, flavonoid biosynthesis, and phenylalanine, tyrosine and tryptophan biosynthesis showed specific significant differential enrichment in wheat in response to *Bgt*-induced stress, whereas carotenoid biosynthesis and carbon fixation in photosynthetic organisms showed the opposite differential performance in response to *Pst*-induced stress (Table 2). The analysis of KEGG pathways showed that 11 specific pathways responded to *Bgt* infection and four specific pathways responded to *Pst* infection, although the *P* values were not significant for those present in the KEGG database (Additional file 1: Table S6). Under *Pst*-induced stress, no DEGs were detected in the mismatch repair, non-homologous end-joining, nucleotide excision repair, DNA replication, and RNA polymerase pathways, which are all involved in genetic information processing. Moreover, ABCB1 (K05658) in ABC transporters, mitochondrial trans-2-enoyl-CoA reductase (K07512) in fatty acid elongation, lipoyl (octanoyl) transferase (K03801) in lipoic acid metabolism, biotin synthase (K01012) in biotin metabolism, glutamate decarboxylase, gamma-glutamyl transpeptidase and cysteamine dioxygenase in taurine and hypotaurine metabolism, and N-glycan biosynthesis were specifically enriched in response to *Bgt* inoculation. The number of dysregulation enzymes reaches to eight in N-glycan biosynthesis especially. In addition, seven distinct KEGG pathways were retrieved with MAS3.0 after comparative analysis of genes differentially expressed in response to *Pst*- and *Bgt*-induced stress, namely nitrogen metabolism, diterpenoid biosynthesis, stilbenoid, diarylheptanoid and gingerol biosynthesis, valine, leucine and isoleucine degradation, photosynthesis, benzoxazinoid biosynthesis, and ribosome pathway. Genes encoding components of conserved protein complexes, such as the proteasome and ribosome, are coexpressed in other organisms and show very similar expression profiles [14]. Surprisingly, half of the enzymes (70 out of 143) showed dysregulated expression in the ribosome pathway in response to *Bgt*, whereas 32 dysregulated enzymes were detected in response to *Pst*-induced stress (Additional file 1: Figure S4). This result indicated that the ribosome pathway is one of the most complex and important pathways regulating genes antagonistic to fungal infection, and that further differential internal remodeling in response to different fungal signals is required.

This analysis showed that the nine functional pathways with *P*-values <0.05 after FDR correction were significantly associated with resistance to fungal infection, whereas the stimulus-specific responses were more robust and 16 pathways included DEGs in comparison of the *Pst* and *Bgt* treatments at 1, 2, and 3 dpi (Additional file 1: Table S5). These results further confirmed the differences in gene regulation and pathways involved in wheat–*Pst* interaction from those involved in wheat–*Bgt* interaction.

### Identification of DEGs shared by *Pst*and *Bgt*infection responses

From aforementioned information, a considerable number of DEGs would have overlapped between the *Bgt* and *Pst* inoculation treatments. Those genes that were significantly regulated in the defense response to both fungi were identified from among the 1,682 overlapping DEGs with annotated information and are listed in Table 3. The overlapping DEGs at each time point are shown in a Venn diagram (Additional file 1: Figure S5). These genes were annotated into 30 GO biological processes with *P*-values <0.001 and considerably dysregulated gene numbers. Protein tetramerization, oxidation-reduction process, xenobiotic metabolic process, response to hypoxia, response to abiotic stimulus, starch biosynthetic process, and cholesterol biosynthetic process were not only significantly enriched but also showed a higher number of dysregulated genes than expected (S/E>1.5) (Table 3). The number of dysregulated genes in the categories of regulation of T cell-mediated cytotoxicity, ketone body biosynthetic process, and ergosterol biosynthetic process was doubled that expected (S/E 8, 4, and 2.4 times, respectively), although there seemed to be a relatively smaller number of DEGs in these three pathways than in the other above-mentioned ones. The GO pedigree analysis indicated that the third main GO hierarchy of biological processes in which the shared genes may participate consisted of response to single-organism stimuli and metabolic process.

**Table 3:** **Go characterization of overlapped DEGs responding to** ***Pst*** **and** ***Bgt*** **stress**

GO.ID	Term	Annotated	Significant	Expected	KS
GO:0009405	Pathogenesis	221	4	6.7	2.30E-16
GO:0036180	Filamentous growth … in response to biotic stimulus	148	3	4.49	1.70E-13
GO:0071216	Cellular response to biotic stimulus	235	5	7.13	6.40E-10
GO:0035690	Cellular response to drug	278	6	8.43	1.60E-08
GO:0006695	Cholesterol biosynthetic process	113	8	3.43	1.10E-07
GO:0055114	Oxidation-reduction process	5917	313	179.4	2.50E-07
GO:0006805	Xenobiotic metabolic process	268	14	8.13	7.70E-06
GO:0006696	Ergosterol biosynthetic process	27	2	0.82	5.80E-05
GO:0010204	Defense response signaling pathway, resistance gene-independent	331	6	10.04	6.90E-05
GO:0045471	Response to ethanol	147	4	4.46	9.00E-05
GO:0030447	Filamentous growth	355	9	10.76	0.00014
GO:0046951	Ketone body biosynthetic process	16	2	0.49	0.00018
GO:0001914	Regulation of T cell mediated cytotoxicity	8	2	0.24	0.00021
GO:0034446	Substrate adhesion-dependent cell spread…	41	1	1.24	0.00021
GO:0001666	Response to hypoxia	637	34	19.31	0.00028
GO:0016045	Detection of bacterium	540	10	16.37	0.00029
GO:0042221	Response to chemical stimulus	17001	693	515.46	0.0003
GO:0006357	Regulation of transcription from RNA pol…	1097	19	33.26	0.00038
GO:0019265	Glycine biosynthetic process, by transam…	15	1	0.45	0.00051
GO:0044182	Filamentous growth of a population of unicellular organisms	272	8	8.25	0.00054
GO:0052548	Regulation of endopeptidase activity	325	8	9.85	0.00054
GO:0006890	Retrograde vesicle-mediated transport, G…	119	3	3.61	0.00055
GO:0009436	Glyoxylate catabolic process	13	1	0.39	0.0007
GO:0043603	Cellular amide metabolic process	466	19	14.13	0.00072
GO:0019252	Starch biosynthetic process	491	56	14.89	0.00076
GO:0010244	Response to low fluence blue light stimu…	20	1	0.61	0.0008
GO:0006144	Purine nucleobase metabolic process	109	5	3.3	0.00085
GO:0044281	Small molecule metabolic process	13733	583	416.38	0.00091
GO:0009628	Response to abiotic stimulus	12227	576	370.71	0.00095
GO:0051262	Protein tetramerization	246	15	7.46	0.00097

**Figure 2:**
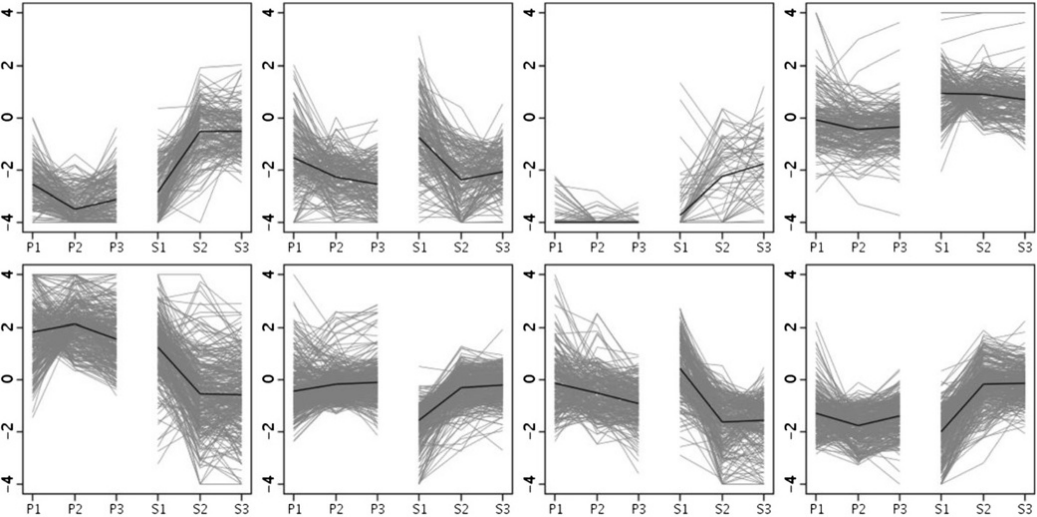
**Clustering.** The expression patterns of 1682 overlapped DE genes for 8 clusters. The horizontal axis indicates each time point and pathogen stress induction condition. The number 1, 2 and 3 mean that N9134 was infected at 1, 2 and 3 dpi, respectively. P represents powdery mildew E09 inoculation condition; S represents stripe rust pathogen CYR 31 inoculation. The vertical axis indicates the log2 fold change calculated between each condition and non-inoculation treatment. Each gray line symbolizes the expression pattern of one gene, and the bold back line illustrates the average expression pattern of all genes in each cluster under *Bgt* or *Pst* stress.

Inspired by similar expression patterns hinting at analogous gene functions, 1,682 genes induced in response to both *Bgt*- and *Pst*-induced stress were screened with a model-based clustering algorithm to identify distinct gene expression profiles among the identified DEGs and to choose a total of eight clusters, to maintain as few tight clusters as possible while including most of the distinct expression patterns (Figure 2). Each gray line in Figure 2 represents the expression pattern for an individual gene, and the single black line indicates the average behavior for induced genes in each cluster. Surprisingly, these overlapping genes showed different expression patterns and complex changes in response to *Pst* infection. This finding illustrated that these genes may perform different functions in response to different stimuli, and implied that wheat triggered distinct genes and regulatory networks to antagonize *Bgt* and *Pst* infection.

Our results for the overlapping DEGs revealed changes in approximately 15% of the transcriptome, with most changes classified as stimulus-specific. This view illustrates the “fluid” nature of the transcriptome and the challenge faced in understanding the complexity of any given stress response.

### Key fungal defense-related genes and pathways

Transcript profiling is capable of revealing pathways of gene expression involved in a defense response, therefore detailed gene-by-gene analysis should be informative because the temporary KEGG pathway and GO annotations were incomplete. A total of 10,583 DEGs were identified, and the range of DEG expression ratios was -235.33 to 187.78 for *Pst* stress-induced genes and -428.85 to 3944.00 for *Bgt* stress-induced genes. To identify the DEGs showing the greatest changes in transcript levels, the top 1% of up- and down-regulated genes in the *Bgt* and *Pst* treatments at each time point (listed in Additional file 1: Table S7) were analyzed.

Comparison of data for N9134 *Pst*- and *Bgt*-inoculated plants with mock-inoculated data revealed gene expression changes that included basal defense transcripts and transcripts specific to the establishment of a biotrophic interaction with fungi. Twelve of the 49 top differentially expressed transcripts significant for treatment were induced by *Pst* inoculation at 1 dpi, whereas 16 were repressed (Additional file 1: Table S7), 12 were down-regulated and three were induced by *Pst* infection at 2 dpi, while eight were repressed and five were up-regulated in response to *Pst* infection at 3 dpi. Ninety-five genes were ranked in the top 1% of genes dysregulated by *Bgt* inoculation, of which 35 were induced and four were repressed at 1 dpi, 23 were up-regulated and 26 were down-regulated at 2 dpi, and 17 were up-regulated and 35 were down-regulated at 3 dpi. As shown in Additional file 1: Table S7, defense-related transcripts accounted for 24.6% (33 out of 134) of the dysregulated transcripts, while 15 (11.2%) were involved in signal transduction, 21 (15.7%) in oxidation-reduction, 8 (6.0%) in biological regulation, 9 (6.7%) in metabolic processes, 5 (3.7%) in protein/carbohydrate transport, 2 in development, 4 (3.0%) in the tricarboxylic acid cycle, 1 in protein modification, and 34 (25.4%) were of unknown/unclear function.

Comparing the top 1% transcriptomes after *Pst* and *Bgt* inoculation, most gene expression patterns showed opposing patterns in the two treatments (Additional file 1: Table S7), especially at the same time point. Those genes induced by *Bgt* infection were usually repressed in the same genotype by *Pst* infection, and vice versa. For example, this pattern was shown by L-type lectin-domain containing receptor kinase IX (T16.19639), hemoglobin 1 (T10.9286), luminal-binding protein (T10.42975), delta-cadinene synthase isozyme XC14 (T13.45944), and hypothetical protein MTR_7g109740 (T4.16776).

### Plants sharing the microbe gene fragments antagonistic to fungal infection

Blast searches of the Nr and Swissport databases showed that 89,672 annotated unigenes matched genes from *Aegilops tauschii* (29.1%), *Triticum urartu* (18.6%), *Hordeum vulgare* (12.9%), *Brachypodium distachyon* (4.7%), *Oryza sativa* (4.4%), and *Marssonina brunnea* (3.4%), while 1565 unigenes (1.7%) were related to *T. aestivum* in the current databases. Interestingly, 285 unigenes matched genes from *Puccinia graminis* (*Pgt*), of which 22 were expressed differentially in the *Pst* treatment, while 33 unigenes were similar to *Apple stem pitting virus* (*ASPV*) genes and 13 were induced in the *Bgt* treatment. A Blast search of the wheat genome with microbial sequences showed that some partial microbial genetic fragments were detected in the wheat genome. For example, a 115 bp fragment of T16_Unigene_BMK.81432 (homologous to arrestin domain-containing protein of *Bgt*) was mapped to chromosomes 7B and 7D with identity of 100% and an E value of 9.0E-57, and a 227 bp fragment of T16_Unigene_BMK.9260 (homologous to hypothetical protein PGTG_00959 of *Pgt*) was mapped to chromosome 1DL with an E value of 1.0E-103.

Ten pairs of gene-specific primers were designed based on *de novo* assembled suspected microbe sequences with the Primer 5.0 software and used in PCRs with genomic DNA extracted from non-infected wheat N9134 leaves as the template. The PCR products showed clear bands, and sequencing confirmed that the two fragments T16_Unigene_BMK.9260 and T17_Unigene_BMK.8064, which were 400 and 280 bp in length, respectively, were amplified from genomic DNA of the resistant wheat germplasm N9134 (Additional file 1: Figure S6). However, most of the checked unigenes could not be verified by sequencing of the PCR products amplified from the genomic DNA, although clear bands were observed in 1.5% agarose gels. This result indicated that most of the unigenes were transcribed from fungi, but some were homologous to wheat genes. Taken together, we inferred that the wheat line shared some genetic material with the microbes, which may be exploited by a virulent fungal pathogen to induce a response directed towards the wrong pathogen.

### High diversity of RGAs

Because of the interest in wheat disease-resistance genes and plant–fungus interactions, the matched disease-resistance-related unigenes were clustered and those unigenes involved in plant–pathogen interaction KEGG pathways were compared between the *Pst* and *Bgt* treatments. In this transcriptome, we detected 2,064 unigenes that matched disease-resistance proteins with BlastX, including 374 unigenes with a NB-ARC conserved domain and 93 containing a NB-LRR domain. Seventy-eight genes were regulated in response to *Bgt* infection of which 62 were induced, whereas 13 unigenes were disturbed by *Pst* inoculation of which only two were induced. Five regulated unigenes were shared by the two fungal stress responses and matched RPM1 and RGA4. This finding indicated that RGAs are highly diverse.

**Figure 3:**
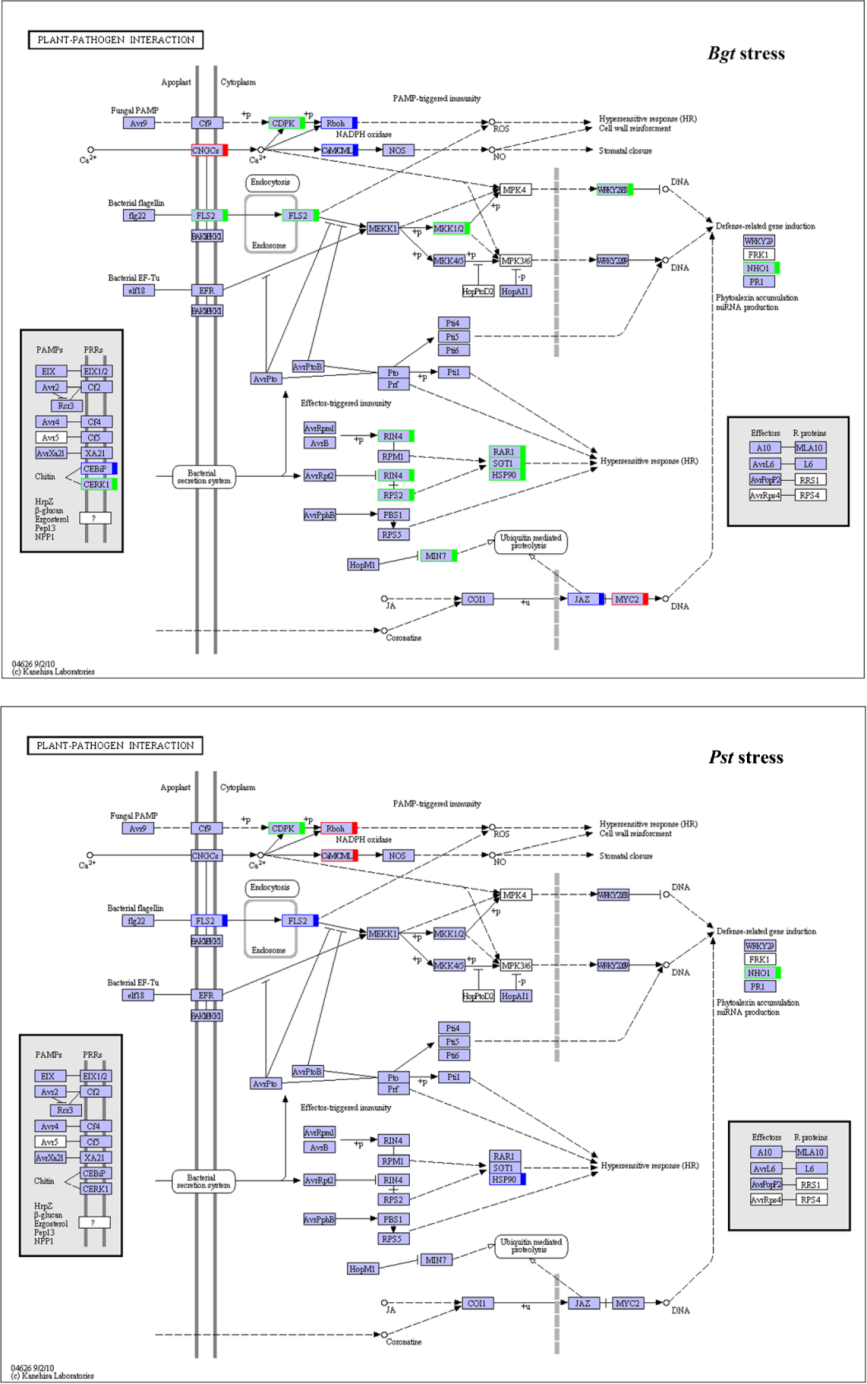
**Differences in disturbed genes matched with enzymes in plant-pathogen interaction pathway between** ***Pst*** **and** ***Bgt*** **infection at 1 dpi.** Green means that the DEGs encoding corresponding enzyme were up regulated in contrasting with non-inoculation; red represent down regulation and blue indicated that DE unigenes are mixed expressed. The diagram of network was cited from KEGG website.

In addition, KEGG enrichment analysis showed that 40 differentially expressed unigenes were associated with plant–fungus interaction pathways and these genes dominated 20 crucial proteins or joints in the *Bgt* stress response, whereas nine differentially expressed unigenes that regulate seven points were detected in the *Pst* stress response. Comparison of the change in expression pattern of these enzymes at 1, 2, and 3 dpi indicated that 1 dpi was the most important time point because nearly all regulated genes were detected at this stage (Figure 3). For resistance to *Pst* infection, wheat manipulated flagellin-sensitive 2 (FLS2) and calcium-dependent protein kinase (CDPK) to trigger the HR response, and induced nonhost 1 (NHO1) at 1 dpi. The respiratory burst oxidase homolog (RBOH, a plasma membrane NADPH oxidase) and heat shock protein (HSP90) were down-regulated. However, the expression levels of FLS2 relative gene was severely decreased compared with that in non-inoculated leaves, whereas NHO1 expression returned to normal at 2 dpi. Perhaps to compensate for the loss, the expression level of HSP90 was induced and attained a level similar to that of CDPK, whereas RBOH expression was restored to normal. At 3 dpi, FLS2, CDPK, and HSP90 genes maintained their respective expression levels observed at 2 dpi at the cost of calmodulin/calmodulin-like (CaM/CML) repression.

In response to *Bgt* inoculation, wheat redeployed most pathways to trigger the HR response at 1 dpi, including PTI, ETI and NHO1. PTI was activated through the reactive oxygen species (ROS) and nitric oxide (NO) signaling pathway mediated by CDPK, RBOH and CaM/CML, FLS2, MKK1/2 (mitogen-activated protein kinase kinase), and WRKY25/33 involved a MAPK cascade. ETI was triggered via RIN4, RPS2, RAR1 (required for Mla12 resistance), SGT1 (suppressor of the G2 allele of SKP1), and HSP90. In addition, MIN7 (guanine nucleotide-exchange factor) and JAZ (jasmonate ZIM domain-containing protein) were induced to activate protein-mediated proteolysis and induce two chitin PRR proteins, Chitin elicitor receptor kinase 1 (CERK1) and Chitin elicitor-binding protein (CEBiP), but started to repress cyclic nucleotide-gated ion channel (CNGC) proteins at 1 dpi. However, most gene induction had ceased at 2 dpi except for six unigenes that matched CaM/CML, MKK1/2, HSP90, NHO1 and MIN7, and three enzymes were repressed, namely RBOH, JAZ and MYC2 as CNGCs. NHO1 and MIN7 continued to be expressed, although the CaM/CML gene and Chitin PRR protein CEBiP were significantly down-regulated at 3 dpi.

Figure 3 shows that wheat accession N9134 employed multi-layered mechanisms to detect and combat pathogens, including preformed physical barriers and physiological and biochemical responses, upon recognition of pathogen-derived elicitors.

## Discussion

In the present study, we performed triplicate deep transcriptome surveys in leaves of the same wheat line inoculated with *Bgt* and *Pst*. Using high-throughput RNA sequencing technology (RNA-Seq), we compared in detail the transcriptional differences and overlap between *Bgt*- and *Pst*-induced stress, and produced currently the most robust and reliable data for investigation of the response of wheat to fungal pathogen attack.

### Identification of differentially expressed genes and comparison with previously reported fungal transcriptome data

Stripe rust and powdery mildew are among the most devastating diseases of wheat in cool regions. Several transcriptome and gene expression analyses of wheat following inoculation with these pathogens have been reported, such as the *Yr5*, *Yr39* high-temperature, adult-plant resistance line versus *Pst* using the Affymetrix GeneChip Wheat Genome Array [15], ‘Shaanmai 139’ versus CYR 32 and ‘Shuiyuan 11’ against CYR 23 using suppression subtractive hybridization and cDNA-AFLP approaches [8]. Similarly, several reports on wheat–*Bgt* interaction have been published. For example, miRNAs and long non-coding RNA regulating the response to *Bgt* infection were analyzed using Solexa high-throughput sequencing [16, 17], and gene expression profiling of wheat in response to *Bgt* infection [18]. Without the requirement for known gene sequences, high-throughput RNA sequencing provides more powerful data for observation of global gene expression profiles in different physiological processes in response to fungal attack than those contained in previous reports. Although we selected the top 1% dysregulated genes as representative, 134 unigenes were listed in Additional file 1: Table S6 owing to the thousands of genes observed in this study. The fungal-induced biological function genes could be divided into nine groups, which covered those reported in previous studies. Defense-related transcripts comprised the biggest group followed by oxidation-reduction, and signal transduction ranked as the third-largest group. The other groups consisted of metabolic, biological regulation, protein/carbohydrate transport, tricarboxylic acid cycle, development, and protein modification, as well as a tenth group categorized as “unknown/unclear”. The results confirmed the expectation, based on observation of resistance-specific transcripts significantly induced at 1–3 dpi, that the top 1% genes were representative of the overall transcriptome. The mass sequence data obtained by deep sequencing of the wheat–fungus interactions provides a robust platform for future functional and molecular research than previously published data.

### Meta-analysis of the KEGG metabolism pathway

In classical genetics, the pathogen-infected host plants and host responses usually showed monogenic control [19]. However, it is probable that thousands of genes can be detected in the early stages of infection, which causes induction of key resistance genes. This means that priming or core control genes must trigger other defense-related and downstream genes. Accordingly, the analysis of pathway enrichment and metabolomics will open up the study of important host–pathogen systems, and may be helpful to identify the important factors that regulate such pathways.

**Figure 4:**
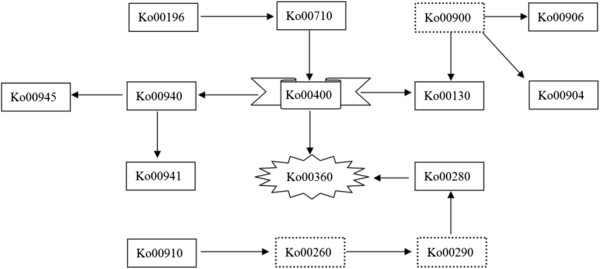
**Overview of a hypothetical metabolism network model constructed with enriched DEGs KEGG pathways according to KEGG annotation.** Aforementioned significant enriched KEE pathways were marked with solid frame, indistinctive KEGG pathways marked with dotted frame. Arrows indicated the fluid direction of metabolic products.

In contrast with previous transcript analyses, we focused mainly on enriched pathways and biological processes in more detail, using meta-analysis of transcripts associated with race-specific pathogen resistance in addition to isolation of early response genes. When responding to pathogen attack, the host plant activates a network of pathways in an endeavor to impair pathogen invasion and to escape damage, which usually includes thousands of genes through a synergistic effect. In addition, bread wheat is hexaploid; as a consequence, characterization of multiple gene copies having mutual or compensatory interactions, such as those encoding the aforementioned disease resistance protein. In this study, we identified seven highly enriched KEGG pathways in response to *Bgt* infection and four in response to *Pst* infection, for example phenylalanine metabolism (Ko00360), phenylalanine, tyrosine and tryptophan biosynthesis (Ko00400), and carbon fixation in photosynthetic organisms (Ko00710). A meta-analysis is advantageous in helping to narrow the field of potential resistance gene analogues and in the search for the critical genes or triggers, and to construct an integrated metabolism network as shown in Figure 4. The final products of the Ko00360 and Ko00945 pathways (stilbenoid, diarylheptanoid and gingerol biosynthesis) are capsaicin and curcumin diglucoside. Capsaicin and curcumin have antibacterial and anticancer properties and reduce inflammatory reactions in mammals [20, 21]; capsaicin also activates serine/threonine kinase and AMP-activated kinase (AMPK), causing increased apoptosis [22]. This may imply that capsaicin and curcumin are similarly employed and processed as antifungal agents in plants, although further physiological and biochemical investigations are needed to test this hypothesis. In addition, this network of pathways will accelerate identification of upstream regulators and key transcription factors.

One component of this network is photosynthesis, and followed by photosynthesis-antenna proteins pathway, which has been reported to modulate plant defense responses induced by pathogen infection and by abiotic signals such as light, circadian rhythm, and temperature [23]. In the present study, 12 enzymes that were dysregulated are participants in the light-harvesting chlorophyll protein complex (LHC) belonging to the photosynthesis-antenna proteins pathway which was significantly enriched in both *Bgt* and *Pst* treatments. Modulation acts on specific key components of plant resistance, indicating that intricate integration of biotic and abiotic signals occurs.

### Relationship between transcriptome divergence and differences in resistance

Although both *Bgt* and *Pst* are fungi, the number of genes induced by *Bgt* infection was 2.1 times higher than the number induced by *Pst* infection at 1 dpi, while the ratio was more than 5 times higher at 2 and 3 dpi. Genes that show highly correlated levels and temporal patterns of expression are often involved in similar functions or cellular processes [24]. Therefore, we clustered 1,682 overlapping DEGs to assess possible crosstalk between transcripts. Unexpectedly, those genes whose expression pattern was similar under *Bgt* attack showed differing temporal patterns of expression under *Pst* infection. This finding indicated that these genes have multiple or different functional roles in response to different biotic stresses. Considering that the wheat germplasm N9134 harbored two pathogen resistance loci, namely a stripe rust resistance gene on chromosome 1B and a powdery mildew resistance gene on chromosome 5B [25], we hypothesized that this discrepancy results from different loci acting as a trigger or elicitor. Most importantly, this study has identified transcripts associated with multi-gene resistance in wheat N9134, which will be useful in future functional studies to identify relevant disease-responsive genes. This result also dispels the current belief that similar mechanisms are activated in response to *Bgt* and *Pst* infection.

### Microbe genes were shared with the wheat genome

The ability to detect and mount a defense response against a potential pathogen has been paramount to the evolutionary and developmental success of plants. Apart from natural selection, an alternative model of evolution involving symbiotic relationships has been proposed and supported by experimental evidence [19, 26]. Selection may act mutually and may drive parasite evolution and host–parasite co-evolution [27]. Likewise, the evolution of plants has also been shaped by molecular interactions with epiphytic, symbiotic, and pathogenic microbes [28]. RNA–RNA recombination is considered to be one of the strongest forces shaping the genomes of plant RNA viruses [29]. Profiting from gene sequences of rust fungi [30], the present transcriptome analysis found that the microbe genes or genomic hallmarks, including genes of *ASPV* and *Pgt* origin, were shared by wheat and could be activated to respond to fungal attack. Based on present knowledge, a reasonable expectation is that wheat expresses the microbe-activated genes to outcompete or overcome hostile RNA as Huang suggested [31]. Paradoxically, molecular evidence proves that only partial unigenes are regulated by fungi, and the remarkable abundance of microbe-derivative genes in infected tissues cannot be an accurate reflection of resistance responses. A satisfactory explanation for the uneven spatial distribution of microbe genes in different fungus-infected tissues will require fine-tuned analyses taking into account the influence of fungal factors. Nevertheless, our results raise the possibility of genetic communication between plants and microbial pathogens, and further substantiates the previous hypothesis that recombination has occurred not only between viruses but even with host RNAs [29]. These features may represent tradeoff advantages of increased genetic variation independent of sexual recombination for biotrophy. Hence, it may provide a notable example of Dollo’s law. This may explain why powdery mildews, stripe rust and possibly other biotrophic parasites became obligate pathogens.

### Key functional genes in plant-pathogen interaction

Plants have evolved innate immune systems that recognize the presence of potential pathogens and initiate effective defense responses. Accumulation of resistance genes has been reported in the PTI and ETI pathways [4, 32]. There are, however, some notable highlights or differences in resistance mechanisms against *Pst* and *Bgt* in wheat. First, previous observations indicated that CNGCs, which are members of the superfamily of ion channels with six transmembrane domains, are involved in diverse physiological functions and have an IQ domain [33, 34], such as AtCNGC2 and AtCNGC4 responding to pathogen attack [35]. In the present study, the transcriptome analysis indicated that CNGC4 was repressed in wheat from 1 to 3 days after infection with the avirulent *Bgt* E09, whereas transcription of 19 genes similar to CNGC1, 5, 8, 14, 15, 17, and 20 were not disturbed in the wheat–pathogen interaction process. In addition, CaM/CML was markedly down-regulated after fungal inoculation, especially at 3 dpi. Although much evidence indicates that deregulation of CaM/CML gene expression or loss of CaM/CML function strongly affects immune responses [36], this change in resistance phenotype was not observed in N9134. This finding indicated that bread wheat synthetically used multiple pathways to perceive and fight fungal infection in addition to the NOS signal pathway. Inspired by the function of DND2, a cyclic nucleotide-gated ion channel reported to accelerate cell death [35], we hypothesize that CNGC4 may play a similar role to resist attack by the powdery mildew pathogen because of the perfect ‘no death phenotype’ resistance of N9134 to *Bgt* E09.

Second, plant CDPKs are a key regulator of innate immune responses to pathogen-associated molecular pattern stimulation [37, 38], and a mutual activation circuit consisting of CPK5 and NADPH oxidase RBOH facilitates rapid signal propagation in the plant [39]. In the current study, we detected one CDPK gene in wheat that was positively persistent and four-fold regulated at all time points (1, 2, and 3 dpi) in response to *Pst* attack, whereas three CDPKs were induced only at 1 dpi. Intriguingly, we found that the RBOH was selectively repressed or induced in the early stages of fungal attack, which provides a chance to identify *in vivo* substrates to unravel CDPK functions and correlate CDPKs with their corresponding substrates to establish their biological significance.

The leucine-rich repeat receptor kinases FLS2 contribute to resistance against bacterial infection in *Arabidopsis thaliana* [40]. The third notable difference in resistance mechanisms against *Pst* and *Bgt* infection in wheat was that the similar serine/threonine-protein kinase FLS2 genes were implicated in protecting wheat seedlings from infection by the two fungi. However, no flg22 or homolog was detected among the unigenes. Our results indicated that the elicitation of this basal immune response is an effective strategy for protecting the plant from both bacterial and fungal pathogens in wheat as well as *Arabidopsis*. We speculate that in wheat an alternative elicitor must interact with FLS2 to contribute to plant resistance.

## Conclusion

The findings presented here provide evidence to clarify the hypothesis that wheat antagonizes stripe rust and powdery mildew infection with similar molecular mechanisms and gene transcripts because both pathogens are biotrophic fungi. In contrast, we found that a disease-resistant wheat line triggers various defense mechanisms to strengthen disease resistance, and that expression patterns of the same defense-associated genes were altered in adaptation to different pathogens. Transcripts of the infection-induced wheat genes were more abundant in *Bgt*-infected leaves than in *Pst*-infected leaves at an early stage following inoculation, especially of genes in plant–pathogen interaction pathways. Our experiment showed that the wheat line shared some microbial genetic materials with the pathogens, which may be exploited to resist infection by a virulent fungal pathogen. Our study provides new insights into the underlying mechanisms related to modulation and regulation of various biochemical pathways in response to fungal infection. Moreover, we provide a powerful platform for further exploration of disease-resistance genes, gene function, molecular research on wheat–fungus interactions, and marker development for classical genetics.

## Methods

### Fungus and plant materials

The winter wheat line N9134, developed at Northwest A&F University, is a line that shows high resistance to *Pst* races CYR 29 and CYR 31 and is resistant to all *Bgt* races in China. This high level of resistance to *Pst* and *Bgt* is conferred by two all-stage resistance genes located on chromosomes 1B and 5BL, respectively. The *Pst* race CYR 31 was maintained by the College of Plant Protection of Northwest A&F University. The *Bgt* isolate E09 was maintained on susceptible wheat ‘Shaanyou 225’. The N9134 plants were cultivated in soil in a growth chamber at 18°C under a 16 h light/8 h dark photoperiod. Half of the 7-day-old seedlings were inoculated with *Bgt* conidia from ‘Shaanyou 225’ seedlings infected 10 days previously. The other halves of the seedlings were inoculated with *Pst* race CYR 31. ‘Shaanyou 225’ and ‘Huixianhong’ were inoculated with E09 and CYR 31 to check that inoculation was successful or unsuccessful, respectively. The inoculated leaves of N9134 were harvested at 0, 1, 2, and 3 dpi, frozen immediately in liquid nitrogen, and stored at -80°C. The test was carried out with three biological replications.

### EST library construction and sequencing

Total RNA was extracted from samples of fungal-inoculated leaves at the specified time points using the TRIzol reagent (BioFlux, Hang Zhou) method with a few modifications pertaining to DNase digestion and RNA purification. A small fraction of the RNA was electrophoresed in a 1% agarose gel to check its quality. Oligo(dT)-magnetic beads were used to enrich the mRNA, which was then broken into fragments with fragmentation buffer. First-strand cDNA was prepared using a reverse transcription-PCR system (Promega, Madison, WI, USA) with random hexamers. Second-strand cDNA was synthesized using RNase H, DNA polymerase I and dNTPs. Poly(A) and adaptor sequences were ligated to the ends of the repaired double-stranded cDNA after purification with a QiaQuick PCR kit. EST libraries were constructed by PCR amplification after checking the quality with agarose gel electrophoresis and sequenced with an Illumina HiSeq™ 2000 platform by Biomarker Technology Co., Ltd (Beijing, China).

### Sequence processing and unigene library

After sequencing, paired-end reads were checked and scored according to the CycleQ20 level standard (i.e., a base quality greater than 20 and an error probability of 0.01). After removinging low-quality reads, all reliable reads were assembled using the Trinity platform to reconstruct a unigene library for the wheat resistance line N9134 [41], and DEG analysis was performed with the bioconductor package DESeq [42]. Gene annotation and pathway identification were performed in accordance with the method described by Shi [43]. To organize genes into hierarchical categories and uncover gene regulatory networks on the basis of biological process, molecular function and cellular components, the DEGs were mapped to Gene Ontology (GO) terms and Kyoto Encyclopedia of Genes and Genomes (KEGG) pathways using the MAS molecular function annotation system (http://bioinfo.capitalbio.com/mas, 11 March 2012). GO terms and KEGG pathways with FDR-corrected *P*-values <0.001 were considered statistically significant. The RPKM values were used to examine the gene expression level distribution for each gene in each sample. In addition, the correlation coefficients (R^2^) between replicates were calculated.

### Quantitative real-time PCR analysis

The SYBR Green Premix Ex Taq™ II quantitative PCR system (Takara, Dalian) was used for qPCR analysis. All experiments involving q-PCR were performed on a 7300 Real-Time PCR System (Applied Biosystems, Foster City, CA, USA) using primers described in Additional file 1: Table S8. The RNA samples used as templates for RNA-Seq were the same as those used for qPCR. The tubulin gene was used as the internal control for normalization of qPCR data. Pearson correlation coefficients between the RNA-Seq and qPCR methods were calculated for eight selected genes across three time points under the stress treatment conditions, based on the average log2 fold change of three biological replicates. PCR was conducted according to the protocol described by Zhang et al. [44].

### Virus genes checked with wheat genome

DNA extraction was carried out using the CTAB method from healthy leaves of wheat line N9134 seedlings. PCR amplifications were performed in total volume of 20 μl following standard protocols. The amplification products were visualized on a 1.5% agarose gel. The microbe gene-specific primers used in the PCRs were designed based on unigene sequences with Primer 5.0 software and synthesized by Beijing AuGCT DNA-SYN Biotechnology Co. (Beijing, China). The primers used are listed in Additional file 1: Table S8.

## Electronic supplementary material

Additional file 1: **The sequence data from this study have been submitted to NCBI** http://http://www.ncbi.nlm.nih.gov **under accession No. PRJNA243835.** **Figure S1.** Evaluate the reliability of RNA-Seq with qRT-PCR. **Figure S2.** MA scatter plot of gene expression level distributions for each treatment comparing to contrast. **Figure S3.** Volcano scatter plot of gene expression level distributions for *Bgt* treatment comparing to *Pst*. **Figure S4a.** Disturbedgenes matched with enzymes of ribosome pathway in *Bgt* infection. **Figure S4b.** Disturbedgenes matched with enzymes of ribosome pathway in *Pst* infection. **Figure S5.** Venn diagram to illustrate the number of DEGs shared by *Pst* and *Bgt*. **Figure S6.** PCR amplification verifies the fragments of microbe in gDNA of N9134. **Table S1.** Statistic correlation coefficients of biological replicates. **Table S2.** GO category (biological process) hits based on the algal functional annotation tool. **Table S3.** GO category (molecular function) hits based on the algal functional annotation tool. **Table S4.** GO category (cellular component) hits based on the algal functional annotation tool. **Table S5.** Significant KEGG enrichment pathway in responses to *Pst* and *Bgt* compared with each other. **Table S6.** KEGG pathway of stimulus-specific responses on *Pst* and *Bgt* compared with non-inoculation. **Table S7.** The detail of the top 1% up and down regulated DEGs in responding to fungi stress at each time points. **Table S8.** Primer sequences used in qRT-PCR with cDNA and PCR with gDNA. (PDF 3 MB)
