# Supplementary material for: Large-scale transcriptome comparison reveals distinct gene activations in wheat responding to stripe rust and powdery mildew
Source: BMC Genomics. 2014 Oct 15;15(1):898. doi: 10.1186/1471-2164-15-898 (PMC4201691; doi:10.1186/1471-2164-15-898)
Supplement: Supplementary file 1 — Additional file 1: The sequence data from this study have been submitted to NCBI http://http://www.ncbi.nlm.nih.gov under accession No. PRJNA243835. Figure S1. Evaluate the reliability of RNA-Seq with qRT-PCR. Figure S2. MA scatter plot of gene expression level distributions for each treatment comparing to contrast. Figure S3. Volcano scatter plot of gene expression level distributions for Bgt treatment comparing to Pst. Figure S4a. Disturbedgenes matched with enzymes of ribosome pathway in Bgt infection. Figure S4b. Disturbedgenes matched with enzymes of ribosome pathway in Pst infection. Figure S5. Venn diagram to illustrate the number of DEGs shared by Pst and Bgt. Figure S6. PCR amplification verifies the fragments of microbe in gDNA of N9134. Table S1. Statistic correlation coefficients of biological replicates. Table S2. GO category (biological process) hits based on the algal functional annotation tool. Table S3. GO category (molecular function) hits based on the algal functional annotation tool. Table S4. GO category (cellular component) hits based on the algal functional annotation tool. Table S5. Significant KEGG enrichment pathway in responses to Pst and Bgt compared with each other. Table S6. KEGG pathway of stimulus-specific responses on Pst and Bgt compared with non-inoculation. Table S7. The detail of the top 1% up and down regulated DEGs in responding to fungi stress at each time points. Table S8. Primer sequences used in qRT-PCR with cDNA and PCR with gDNA. (PDF 3 MB) [file 12864_2014_6560_MOESM1_ESM.pdf]

Table S1 Statistic correlation coefficients of biological replicates using to assemble sequences

**Sample**	**test**	**Correlation**	***P*-value**
**S1**	R1-vs-R2	0.9932625	2.20E-16
	R1-vs-R3	0.9843602	2.20E-16
	R2-vs-R3	0.982757	2.20E-16
**S2**	R1-vs-R3	0.9408083	2.20E-16
**S3**	R1-vs-R2	0.9940531	2.20E-16
	R1-vs-R3	0.9920043	2.20E-16
	R2-vs-R3	0.991774	2.20E-16
**P1**	R1-vs-R2	0.9832744	2.20E-16
**P2**	R2-vs-R3	0.9834377	2.20E-16
**P3**	R1-vs-R2	0.9850565	2.20E-16
	R1-vs-R3	0.9296429	2.20E-16
	R2-vs-R3	0.9445614	2.20E-16
**Ck**	R1-vs-R2	0.9357648	2.20E-16
	R1-vs-R3	0.9659491	2.20E-16
	R2-vs-R3	0.9887152	2.20E-16

Note: Ck: resistant wheat N9134 exempt from pathogen stress; S1, S2 and S3: N9134 infected with stripe rust pathogen CYR 31 at 1, 2 and 3 dpi, respectively; P1, P2 and P3: N9134 infected with powdery mildew pathogen E09 at 1, 2 and 3 dpi, respectively.

Table S2 Characterization of annotated Go term (biological process) Unigenes induced by stripe rust pathogen and powdery mildew pathogen.

GO.ID	Term	Annotated Unigenes	*Bgt* stress only DEGs			Overlapped DEGs			*Pst* stress only DEGs		
			Significant	Expected	KS	Significant	Expected	KS	Significant	Expected	KS
GO:0043581	mycelium development	336	38	42.16	< 1e-30	0	10.19	1.00E-30	1	16.92	1.00E-30
GO:0006278	RNA-dependent DNA replication	2024	28	253.97	9.60E-25	6	61.37	1.00E-30	10	101.93	1.00E-30
GO:0006310	DNA recombination	2260	64	283.59	8.00E-20	16	68.52	4.80E-29	26	113.81	1.80E-26
GO:0009405	pathogenesis	221	31	27.73	1.40E-14	4	6.7	2.30E-16	6	11.13	6.80E-16
GO:0055114	oxidation-reduction process	5917	966	742.47	7.10E-14	313	179.4	2.50E-07	431	297.98	8.50E-08
GO:0015074	DNA integration	1325	9	166.26	3.00E-12	0	40.17	3.20E-19	0	66.73	8.00E-17
GO:0036180	filamentous growth of a population of un...	148	18	18.57	1.80E-11	3	4.49	1.70E-13	6	7.45	1.70E-13
GO:0036170	filamentous growth of a population of un...	111	11	13.93	2.00E-09	1	3.37	1.40E-11	4	5.59	7.20E-12
GO:0006805	xenobiotic metabolic process	268	51	33.63	6.40E-09	14	8.13	7.70E-06	15	13.5	7.40E-05
GO:0080167	response to karrikin	869	195	109.04	1.70E-08	67	26.35	0.01662	89	43.76	0.00644
GO:0009751	response to salicylic acid stimulus	2504	478	314.2	2.40E-08	111	75.92	0.02462	157	126.1	0.02352
GO:0035690	cellular response to drug	278	39	34.88	4.80E-08	6	8.43	1.60E-08	9	14	2.70E-08
GO:0009737	response to abscisic acid stimulus	3359	596	421.49	7.00E-08	157	101.84	0.1422	230	169.16	0.09324
GO:0015706	nitrate transport	929	203	116.57	7.20E-08	50	28.17	0.35023	61	46.78	0.63822
GO:0010167	response to nitrate	924	202	115.94	7.60E-08	52	28.02	0.28015	62	46.53	0.58558
GO:0071216	cellular response to biotic stimulus	235	24	29.49	2.30E-07	5	7.13	6.40E-10	11	11.83	7.30E-10
GO:0006956	complement activation	55	4	6.9	8.30E-07	0	1.67	5.40E-09	0	2.77	1.80E-08
GO:0009725	response to hormone stimulus	8354	1404	1048.26	8.90E-07	372	253.29	0.00961	579	420.7	0.00024
GO:0006695	cholesterol biosynthetic process	113	23	14.18	9.30E-07	8	3.43	1.10E-07	12	5.69	6.70E-08
GO:0044283	small molecule biosynthetic process	7307	1308	916.89	1.50E-06	350	221.54	0.00793	488	367.98	0.04628
GO:0009723	response to ethylene stimulus	1757	325	220.47	1.70E-06	80	53.27	0.2758	119	88.48	0.25493
GO:0009628	response to abiotic stimulus	12227	1961	1534.25	1.80E-06	576	370.71	0.00095	835	615.75	0.00361
GO:0009813	flavonoid biosynthetic process	1356	286	170.15	1.90E-06	82	41.11	0.0635	102	68.29	0.1485

GO:0010264	myo-inositol hexakisphosphate biosynthet...	138	47	17.32	2.60E-06	20	4.18	0.02618	25	6.95	0.00867
GO:0006415	translational termination	871	86	109.29	2.70E-06	3	26.41	2.80E-13	26	43.86	7.40E-15
GO:0006614	SRP-dependent cotranslational protein ta...	866	95	108.67	2.80E-06	4	26.26	2.40E-12	29	43.61	1.70E-14
GO:0044249	cellular biosynthetic process	24850	3395	3118.19	2.90E-06	858	753.44	0.01258	1395	1251.44	0.00075
GO:0009755	hormone-mediated signaling pathway	4164	676	522.5	3.30E-06	167	126.25	0.22796	258	209.7	0.2032
GO:0006979	response to oxidative stress	3078	546	386.23	4.20E-06	178	93.32	0.00461	230	155.01	0.01407
GO:0006863	purine nucleobase transport	235	67	29.49	4.30E-06	17	7.13	0.35396	23	11.83	0.30243
GO:0010363	regulation of plant-type hypersensitive ...	1880	340	235.9	4.40E-06	68	57	0.73254	102	94.68	0.64029
GO:0010200	response to chitin	1773	321	222.48	4.50E-06	67	53.76	0.01735	106	89.29	0.01192
GO:0046148	pigment biosynthetic process	1542	288	193.49	5.90E-06	95	46.75	0.02655	134	77.65	0.0072
GO:0002679	respiratory burst involved in defense re...	560	125	70.27	8.10E-06	20	16.98	0.09007	35	28.2	0.04024
GO:0009620	response to fungus	3158	530	396.27	8.80E-06	114	95.75	0.15934	179	159.04	0.14345
GO:0080052	response to histidine	80	31	10.04	9.30E-06	7	2.43	0.55701	8	4.03	0.6232
GO:0080053	response to phenylalanine	80	31	10.04	9.30E-06	7	2.43	0.55701	8	4.03	0.6232
GO:0009611	response to wounding	3056	479	383.47	1.30E-05	116	92.66	0.40113	169	153.9	0.4371
GO:0080009	mRNA methylation	39	10	4.89	1.40E-05	0	1.18	1.10E-05	0	1.96	3.30E-05
GO:0017187	peptidyl-glutamic acid carboxylation	12	1	1.51	1.50E-05	0	0.36	1.70E-06	0	0.6	3.30E-06
GO:0006414	translational elongation	1105	96	138.66	1.80E-05	5	33.5	1.20E-13	34	55.65	2.50E-15
GO:0042430	indole-containing compound metabolic pro..	791	159	99.26	1.80E-05	39	23.98	0.39937	52	39.83	0.51525
GO:0006612	protein targeting to membrane	2730	424	342.56	2.40E-05	74	82.77	0.77184	134	137.48	0.70544
GO:0015804	neutral amino acid transport	363	86	45.55	2.60E-05	39	21.92	0.36391	57	36.41	0.28016
GO:0009624	response to nematode	723	151	90.72	2.60E-05	20	11.01	0.19712	24	18.28	0.36282
GO:0044281	small molecule metabolic process	13733	2135	1723.22	3.40E-05	583	416.38	0.00091	859	691.59	0.00132
GO:0019083	viral transcription	890	85	111.68	4.20E-05	4	26.98	2.30E-11	26	44.82	2.40E-12
GO:0042538	hyperosmotic salinity response	1016	205	127.49	4.40E-05	60	30.8	0.16328	79	51.17	0.19171
GO:0006552	leucine catabolic process	20	12	2.51	4.70E-05	8	0.61	0.0033	8	1.01	0.00504

**GO:0000184**	**nuclear-transcribed mRNA catabolic proce...**	915	93	114.81	5.70E-05	6	27.74	7.20E-11	29	46.08	4.10E-12
**GO:0006526**	**arginine biosynthetic process**	35	9	4.39	6.60E-05	0	1.06	0.00013	0	1.76	0.00023
**GO:0042631**	**cellular response to water deprivation**	407	92	51.07	7.70E-05	67	53.15	0.14457	43	20.5	0.04303
**GO:0009863**	**salicylic acid mediated signaling pathwa...**	1753	305	219.97	7.70E-05	34	12.34	0.05947	97	88.28	0.15181
**GO:0043201**	**response to leucine**	88	31	11.04	8.00E-05	7	2.67	0.61723	8	4.43	0.69021
**GO:0030050**	**vesicle transport along actin filament**	16	2	2.01	8.10E-05	0	0.49	0.00091	0	0.81	0.00134
**GO:0042594**	**response to starvation**	1698	303	213.07	9.20E-05	85	51.48	0.04704	110	85.51	0.10049
**GO:0071705**	**nitrogen compound transport**	3535	660	443.57	9.60E-05	143	107.18	0.09566	205	178.02	0.20737
**GO:0044416**	**induction by symbiont of host defense re...**	48	11	6.02	9.80E-05	0	1.46	0.00018	2	2.42	0.00011
**GO:0006396**	**RNA processing**	5438	769	682.36	0.0001	174	164.88	0.08411	322	273.86	0.00602
**GO:0019448**	**L-cysteine catabolic process**	15	0	1.88	0.00012	0	0.45	6.30E-06	0	0.76	1.30E-05
**GO:0010107**	**potassium ion import**	57	23	7.15	0.00014	11	1.73	0.04247	11	2.87	0.08148
**GO:0046951**	**ketone body biosynthetic process**	16	4	2.01	0.00015	2	0.49	0.00018	2	0.81	0.00028
**GO:0072330**	**monocarboxylic acid biosynthetic process**	2991	495	375.31	0.00016	121	90.69	0.0531	175	150.63	0.10671
**GO:0034976**	**response to endoplasmic reticulum stress**	1610	282	202.02	0.00017	65	60.79	0.56995	87	81.08	0.07479
**GO:0009627**	**systemic acquired resistance**	2005	340	251.59	0.00017	57	48.81	0.08689	102	100.97	0.5391
**GO:0042939**	**tripeptide transport**	143	42	17.94	0.00019	9	4.34	0.62341	11	7.2	0.70775
**GO:0010033**	**response to organic substance**	13139	2157	1648.69	0.0002	541	398.37	0.37611	836	661.67	0.42073
**GO:0006366**	**transcription from RNA polymerase II pro...**	1604	142	201.27	0.00021	29	48.63	0.05446	58	80.78	0.04386
**GO:0009699**	**phenylpropanoid biosynthetic process**	2251	458	282.46	0.00022	133	68.25	0.01741	161	113.36	0.10835
**GO:0065007**	**biological regulation**	24762	3440	3107.15	0.00025	817	750.77	0.20705	1381	1247	0.00556
**GO:0000001**	**mitochondrion inheritance**	13	0	1.63	0.00027	0	0.39	5.30E-05	0	0.65	8.10E-05
**GO:0009963**	**positive regulation of flavonoid biosynt...**	497	106	62.36	0.00029	24	15.07	0.70952	29	25.03	0.74278
**GO:0080024**	**indolebutyric acid metabolic process**	57	20	7.15	0.00032	6	1.73	0.08398	7	2.87	0.08507
**GO:0016036**	**cellular response to phosphate starvatio...**	477	103	59.85	0.00033	33	14.46	0.19724	44	24.02	0.14257
**GO:0043651**	**linoleic acid metabolic process**	26	2	3.26	0.00034	0	0.76	3.60E-05	0	1.26	6.60E-05

GO:0030476	ascospore wall assembly	25	1	3.14	0.00034	4	0.33	0.04099	4	0.55	0.05656
GO:0043496	regulation of protein homodimerization a...	11	8	1.38	0.00034	0	0.79	9.50E-05	0	1.31	0.0002
GO:0044409	entry into host	78	13	9.79	0.00036	0	2.36	0.00033	1	3.93	0.00034
GO:0010114	response to red light	551	115	69.14	0.00037	3	37.99	3.80E-11	35	63.1	2.80E-12
GO:0006413	translational initiation	1253	101	157.23	0.00037	55	16.71	0.00406	74	27.75	0.00032
GO:0031505	fungal-type cell wall organization	104	11	13.05	0.00038	0	3.15	0.00013	3	5.24	1.90E-05
GO:0042938	dipeptide transport	139	40	17.44	0.00038	9	4.21	0.65779	11	7	0.6592
GO:0006103	2-oxoglutarate metabolic process	86	19	10.79	0.00041	4	2.61	0.00734	6	4.33	0.00576
GO:0051502	diterpene phytoalexin biosynthetic proce...	75	25	9.41	0.00042	2	2.27	0.06904	3	3.78	0.07837
GO:0006891	intra-Golgi vesicle-mediated transport	284	54	35.64	0.00044	5	8.61	0.01918	10	14.3	0.03401
GO:0009987	cellular process	45474	5883	5706.1	0.00045	1430	1378.74	0.01375	2370	2290.05	0.00462
GO:0052746	inositol phosphorylation	9	7	1.13	0.00046	0	0.27	0.5797	0	0.45	0.61507
GO:0016114	terpenoid biosynthetic process	1265	240	158.73	0.00048	66	38.35	0.09471	100	63.7	0.05177
GO:0000041	transition metal ion transport	1452	253	182.2	0.00049	56	44.02	0.30982	79	73.12	0.40997
GO:0030447	filamentous growth	355	44	44.55	0.00055	9	10.76	0.00014	1	3.22	9.70E-05
GO:0030448	hyphal growth	64	8	8.03	0.00055	0	1.94	0.00015	14	17.88	0.00027
GO:0006696	ergosterol biosynthetic process	27	3	3.39	0.00056	2	0.82	5.80E-05	2	1.36	0.00011
GO:0009753	response to jasmonic acid stimulus	2460	429	308.68	0.00061	108	74.59	0.1923	161	123.88	0.17187
GO:0051591	response to cAMP	86	6	10.79	0.00067	0	2.61	3.80E-05	0	4.33	0.00018
GO:0007118	budding cell apical bud growth	11	0	1.38	0.00068	0	0.33	0.00017	0	0.55	0.00024
GO:0010183	pollen tube guidance	61	17	7.65	0.0007	2	1.85	0.1031	3	3.07	0.14081
GO:0051262	protein tetramerization	246	41	30.87	0.00075	15	7.46	0.00097	23	12.39	0.00018
GO:0019878	lysine biosynthetic process via aminoadi...	19	0	2.38	0.00077	0	0.73	2.40E-05	0	1.21	5.70E-05
GO:0019373	epoxygenase P450 pathway	24	1	3.01	0.00077	0	0.58	0.00022	0	0.96	0.00031
GO:0010035	response to inorganic substance	7436	1228	933.07	0.00083	150	83.14	0.02452	492	374.47	0.01069
GO:0006790	sulfur compound metabolic process	2742	467	344.07	0.00083	348	225.45	0.00725	197	138.09	0.05135

GO:0000727	double-strand break repair via break-ind...	6	0	0.75	0.00085	0	0.18	0.00016	0	0.3	0.00024
GO:0010099	regulation of photomorphogenesis	86	28	10.79	0.00085	8	2.61	0.46987	10	4.33	0.41732
GO:0010324	membrane invagination	132	9	16.56	0.00086	1	4	0.10736	3	6.65	0.11838
GO:0006690	icosanoid metabolic process	143	29	17.94	0.00088	5	4.34	0.10015	8	7.2	0.1506
GO:0044182	filamentous growth of a population of un...	272	38	34.13	0.00092	0	3	0.00026	3	4.99	0.00011
GO:0044011	single-species biofilm formation on inan...	99	10	12.42	0.00092	8	8.25	0.00054	13	13.7	0.00154
GO:0016101	diterpenoid metabolic process	528	119	66.25	0.00096	109	53.73	0.01457	167	89.24	0.03966
GO:0008299	isoprenoid biosynthetic process	1772	342	222.35	0.00096	26	16.01	0.08673	33	26.59	0.1687
GO:0019344	cysteine biosynthetic process	831	155	104.27	0.001	55	25.2	0.08786	65	41.85	0.22009
GO:0019252	starch biosynthetic process	491	97	61.61	0.00534	56	14.89	0.00076	74	24.73	3.50E-05
GO:0006897	endocytosis	1244	83	156.1	0.0665	12	37.72	7.80E-05	38	62.65	6.20E-05
GO:0030307	positive regulation of cell growth	109	10	13.68	0.00172	2	3.3	4.60E-05	3	5.49	6.70E-05
GO:0045471	response to ethanol	147	17	18.45	0.003	4	4.46	9.00E-05	7	7.4	7.80E-05
GO:0070374	positive regulation of ERK1 and ERK cas...	17	2	2.13	0.01851	0	0.52	0.00207	2	0.86	8.50E-05
GO:0006450	regulation of translational fidelity	70	5	8.78	0.00296	0	2.12	0.0001	2	3.53	9.50E-05
GO:0010204	defense response signaling pathway, resi...	331	26	41.53	0.00369	6	10.04	6.90E-05	13	16.67	0.0001
GO:0032007	negative regulation of TOR signaling cas...	11	1	1.38	0.00308	0	0.33	0.00086	1	0.55	0.00011
GO:0090304	nucleic acid metabolic process	19234	2080	2413.49	0.07559	486	583.16	0.34502	910	968.62	0.00011
GO:0032506	cytokinetic process	175	21	21.96	0.0056	1	5.31	0.00144	10	8.81	0.00013
GO:0009792	embryo development ending in birth or eg...	1646	141	206.54	0.35607	17	49.91	0.00027	53	82.89	0.00014
GO:0019067	viral assembly, maturation, egress, and ...	74	6	9.29	0.00397	1	2.24	0.00015	3	3.73	0.00015
GO:0042738	exogenous drug catabolic process	26	0	3.26	0.00217	0	0.79	7.30E-05	0	1.31	0.00017
GO:0061178	regulation of insulin secretion involved...	19	1	2.38	0.00152	0	0.58	0.00348	0	0.96	0.0002
GO:0051046	regulation of secretion	312	32	39.15	0.00229	2	9.46	0.03258	7	15.71	0.0002
GO:0030435	sporulation resulting in formation of a ...	130	8	16.31	0.04477	1	3.94	0.00028	2	6.55	0.00021
GO:0010244	response to low fluence blue light stimu...	20	7	2.51	0.00116	1	0.61	0.0008	2	1.01	0.00023

GO:0006283	transcription-coupled nucleotide-excisio...	77	1	9.66	0.00146	0	2.33	0.00014	0	3.88	0.00027
GO:0042760	very long-chain fatty acid catabolic pro...	12	0	1.51	0.00109	0	0.36	0.00017	0	0.6	0.00028
GO:0000023	maltose metabolic process	496	86	62.24	0.08399	52	15.04	0.00229	68	24.98	0.00029
GO:0001914	regulation of T cell mediated cytotoxici...	8	2	1	0.00131	2	0.24	0.00021	2	0.4	0.00032
GO:0034418	urate biosynthetic process	13	0	1.63	0.00101	0	0.39	0.00022	0	0.65	0.00033
GO:0043603	cellular amide metabolic process	466	65	58.47	0.00658	19	14.13	0.00072	31	23.47	0.00034
GO:0019288	isopentenyl diphosphate biosynthetic pro...	634	112	79.55	0.02345	49	19.22	0.04131	80	31.93	0.00035
GO:0016045	detection of bacterium	540	38	67.76	0.0107	10	16.37	0.00029	20	27.19	0.00036
GO:0030449	regulation of complement activation	27	3	3.39	0.00132	0	0.82	0.00017	0	1.36	0.00038
GO:0007596	blood coagulation	427	38	53.58	0.02328	5	12.95	7.50E-05	8	21.5	0.00038
GO:0042364	water-soluble vitamin biosynthetic proce...	167	30	20.96	0.01138	10	5.06	0.00515	16	8.41	0.00041
GO:1901700	response to oxygen-containing compound	10514	1780	1319.3	0.03053	480	318.78	0.00146	703	529.48	0.00042
GO:0001403	invasive growth in response to glucose l...	6	1	0.75	0.00141	0	0.18	0.00031	0	0.3	0.00044
GO:0006144	purine nucleobase metabolic process	109	11	13.68	0.00153	5	3.3	0.00085	7	5.49	0.00044
GO:0006418	tRNA aminoacylation for protein translat...	245	18	30.74	0.00332	1	7.43	0.00112	8	12.34	0.00045
GO:0000011	vacuole inheritance	21	1	2.64	0.00197	0	0.64	0.00031	0	1.06	0.00051
GO:0048732	gland development	216	15	27.1	0.02019	4	6.55	0.00493	5	10.88	0.00052
GO:0034446	substrate adhesion-dependent cell spread...	41	4	5.14	0.0097	1	1.24	0.00021	1	2.06	0.00057
GO:0070989	oxidative demethylation	36	1	4.52	0.00533	0	1.09	0.00029	0	1.81	0.00063
GO:0060627	regulation of vesicle-mediated transport	305	27	38.27	0.00876	3	9.25	0.0073	7	15.36	0.00065
GO:0043967	histone H4 acetylation	108	9	13.55	0.00298	1	3.27	0.00068	3	5.44	0.00071
GO:0000750	pheromone-dependent signal transduction...	7	0	0.88	0.00189	0	0.21	0.00055	0	0.35	0.00075
GO:0009658	chloroplast organization	619	107	77.67	0.04995	43	18.77	0.12431	77	31.17	0.00076
GO:0048313	Golgi inheritance	18	1	2.26	0.01186	0	0.55	0.00281	1	0.91	0.00077
GO:0006370	7-methylguanosine mRNA capping	63	0	7.91	0.00327	0	1.91	0.00044	0	3.17	0.00078
GO:0033129	positive regulation of histone phosphory...	6	1	0.75	0.02623	0	0.18	0.00943	1	0.3	0.00078

GO:0045038	protein import into chloroplast thylakoi...	15	4	1.88	0.49237	4	0.45	0.17554	8	0.76	0.00078
GO:0006890	retrograde vesicle-mediated transport, G...	119	19	14.93	0.00158	3	3.61	0.00055	4	5.99	0.00079
GO:0018279	protein N-linked glycosylation via aspar...	179	17	22.46	0.00116	2	5.43	0.00027	4	9.01	0.00084
GO:0030466	chromatin silencing at silent mating-typ...	10	0	1.25	0.0021	0	0.3	0.00062	0	0.5	0.00086
GO:0035066	positive regulation of histone acetylati...	10	2	1.25	0.01588	0	0.3	0.00579	1	0.5	0.00086
GO:0000082	G1/S transition of mitotic cell cycle	437	28	54.84	0.01177	3	13.25	0.0002	6	22.01	0.00088
GO:0019265	glycine biosynthetic process, by transam...	15	1	1.88	0.00512	1	0.45	0.00051	1	0.76	0.0009
GO:0001666	response to hypoxia	637	100	79.93	0.01894	34	19.31	0.00028	45	32.08	0.00092
GO:0000075	cell cycle checkpoint	594	34	74.54	0.01572	3	18.01	0.00028	11	29.91	0.00093
GO:0031425	chloroplast RNA processing	353	18	44.29	0.00277	0	10.7	0.00041	2	17.78	0.00094
GO:0042221	response to chemical stimulus	17001	2719	2133.3	0.00131	693	515.46	0.0003	1052	856.16	0.04557
GO:0006357	regulation of transcription from RNA pol...	1097	86	137.65	0.01648	19	33.26	0.00038	36	55.24	0.00155
GO:0052548	regulation of endopeptidase activity	325	42	40.78	0.00927	8	9.85	0.00054	8	16.37	0.00627
GO:0030437	ascospore formation	60	3	7.53	0.00164	0	1.82	0.00058	0	3.02	0.00105
GO:0009436	glyoxylate catabolic process	13	1	1.63	0.00582	1	0.39	0.0007	1	0.65	0.00118
GO:1901069	guanosine-containing compound catabolic ...	777	57	97.5	0.09556	7	23.56	0.00078	23	39.13	0.00108
GO:0060628	regulation of ER to Golgi vesicle-mediat...	10	2	1.25	0.00394	0	0.3	0.0008	0	0.5	0.00123
GO:0007107	membrane addition at site of cytokinesis	9	0	1.13	0.00257	0	0.27	0.00081	0	0.45	0.0011
GO:0045033	peroxisome inheritance	9	0	1.13	0.00257	0	0.27	0.00081	0	0.45	0.0011
GO:0031099	regeneration	123	10	15.43	0.01279	2	3.73	0.00084	4	6.19	0.00247
GO:0051298	centrosome duplication	142	10	17.82	0.02957	2	4.31	0.00095	2	7.15	0.0035
GO:0008298	intracellular mRNA localization	78	5	9.79	0.00285	0	2.36	0.00097	1	3.93	0.00184
GO:0051666	actin cortical patch localization	8	1	1	0.00575	0	0.24	0.00099	0	0.4	0.00149
GO:0006706	steroid catabolic process	31	1	3.89	0.00389	0	0.94	0.001	0	1.56	0.00196

Table S3 Characterization of annotated Go term (molecular function) Unigenes induced by stripe rust pathogen and powdery mildew pathogen.

GO ID	Term	Annotated unigenes	*Bgt* stress only DEGs			Overlapped DEGs			*Pst* stress only DEGs		
			Significant	Expected	KS	Significant	Expected	KS	Significant	Expected	KS
GO:0003964	RNA-directed DNA polymerase activity	2050	27	245.79	8.00E-24	6	58.15	< 1e-30	9	97.05	< 1e-30
GO:0005506	iron ion binding	1185	246	142.08	1.00E-13	80	33.61	3.10E-08	98	56.1	7.30E-08
GO:0020037	heme binding	1126	229	135.01	7.30E-08	70	31.94	0.00011	84	53.31	0.00042
GO:0004190	aspartic-type endopeptidase activity	807	34	96.76	3.90E-07	9	22.89	2.20E-10	12	38.2	4.30E-09
GO:0008395	steroid hydroxylase activity	84	16	10.07	2.20E-06	9	2.38	4.30E-07	10	3.98	5.80E-07
GO:0016709	oxidoreductase activity, acting on paire...	571	145	68.46	4.40E-06	45	16.2	2.40E-05	54	27.03	9.80E-07
GO:0016706	oxidoreductase activity, acting on paire...	296	60	35.49	1.10E-05	14	8.4	0.00147	16	14.01	0.00482
GO:0015112	nitrate transmembrane transporter activi...	152	45	18.22	2.60E-05	11	4.31	0.46663	14	7.2	0.4542
GO:0050734	hydroxycinnamoyltransferase activity	77	30	9.23	3.40E-05	7	2.18	0.01288	7	3.65	0.02845
GO:0016772	transferase activity, transferring phosp...	10045	932	1204.38	3.90E-05	189	284.94	1.10E-06	302	475.54	1.60E-05
GO:0015334	high affinity oligopeptide transporter a...	120	38	14.39	6.20E-05	9	3.4	0.59039	11	5.68	0.61955
GO:0008061	chitin binding	69	23	8.27	8.00E-05	5	1.96	0.00503	6	3.27	0.00513
GO:0009055	electron carrier activity	1485	259	178.05	8.90E-05	84	42.12	0.00608	102	70.3	0.02545
GO:0008174	mRNA methyltransferase activity	45	10	5.4	0.0001	0	1.28	4.80E-05	0	2.13	0.00014
GO:0005515	protein binding	19161	2489	2297.38	0.00011	557	543.52	0.00321	1007	907.1	9.30E-05
GO:0036209	9beta-pimara-7,15-diene oxidase activity	49	18	5.88	0.00012	2	1.39	0.03665	2	2.32	0.06335
GO:0015175	neutral amino acid transmembrane transpo	115	36	13.79	0.00013	8	3.26	0.07996	8	5.44	0.15518
GO:0016833	oxo-acid-lyase activity	31	6	3.72	0.00014	3	0.88	0.00026	4	1.47	0.00046
GO:0030170	pyridoxal phosphate binding	380	61	45.56	0.00014	21	10.78	0.00016	26	17.99	0.00034
GO:0003676	nucleic acid binding	12630	939	1514.32	0.00015	215	358.26	1.70E-10	414	597.92	2.20E-15
GO:0008404	arachidonic acid 14,15-epoxygenase activ...	11	1	1.32	0.00019	0	0.31	2.80E-05	0	0.52	4.60E-05
GO:0008405	arachidonic acid 11,12-epoxygenase activ...	11	1	1.32	0.00019	0	0.31	2.80E-05	0	0.52	4.60E-05
GO:0004812	aminoacyl-tRNA ligase activity	253	17	30.33	0.0002	1	7.18	0.00041	8	11.98	0.00014

GO:0080043	quercetin 3-O-glucosyltransferase activi...	238	58	28.54	0.00021	16	6.75	0.34981	18	11.27	0.53262
GO:0042936	dipeptide transporter activity	139	40	16.67	0.00022	9	3.94	0.66493	11	6.58	0.68335
GO:0016165	lipoxygenase activity	65	24	7.79	0.00028	4	1.84	0.69968	4	3.08	0.72289
GO:0043008	ATP-dependent protein binding	24	2	2.88	0.00029	0	0.68	0.00117	0	1.14	0.00179
GO:0016410	N-acyltransferase activity	352	62	42.2	0.0003	16	9.98	0.00303	27	16.66	0.00725
GO:0016616	oxidoreductase activity, acting on the C...	775	131	92.92	0.0003	38	21.98	0.00739	51	36.69	0.01841
GO:0030976	thiamine pyrophosphate binding	38	8	4.56	0.00031	1	1.08	8.90E-05	1	1.8	0.00017
GO:0042937	tripeptide transporter activity	137	39	16.43	0.00033	9	3.89	0.6496	11	6.49	0.68857
GO:0016413	O-acetyltransferase activity	65	20	7.79	0.00036	4	1.84	0.21728	5	3.08	0.23227
GO:0043424	protein histidine kinase binding	81	24	9.71	0.00044	7	2.3	0.04365	8	3.83	0.06613
GO:0004100	chitin synthase activity	23	0	2.76	0.00052	0	0.65	9.30E-05	0	1.09	0.00016
GO:0008236	serine-type peptidase activity	587	72	70.38	0.00062	11	16.65	0.00113	17	27.79	0.0033
GO:0016780	phosphotransferase activity, for other s...	34	5	4.08	0.00067	3	0.96	0.00182	3	1.61	0.0002
GO:0008374	O-acyltransferase activity	308	79	36.93	0.00071	22	8.74	0.00374	25	14.58	0.00799
GO:0004523	ribonuclease H activity	270	0	32.37	0.00074	0	7.66	1.10E-05	0	12.78	3.80E-05
GO:0004672	protein kinase activity	5613	640	672.99	0.00078	124	159.22	1.40E-05	201	265.73	0.00095
GO:0036201	ent-isokaurene C2-hydroxylase activity	84	25	10.07	0.00078	7	2.38	0.00865	8	3.98	0.01971
GO:0005315	inorganic phosphate transmembrane transp	29	12	3.48	0.00081	6	0.82	0.01645	6	1.37	0.0289
GO:0052638	indole-3-butyrate beta-glucosyltransfera...	51	17	6.11	0.00081	5	1.45	0.06666	6	2.41	0.0623
GO:0072341	modified amino acid binding	132	26	15.83	0.00085	8	3.74	0.00784	9	6.25	0.0166
GO:0005338	nucleotide-sugar transmembrane transport.	55	17	6.59	0.00087	2	1.56	0.00033	5	2.6	0.00021
GO:0003735	structural constituent of ribosome	1291	138	154.79	0.00271	16	36.62	5.00E-07	56	61.12	2.00E-09
GO:0034875	caffeine oxidase activity	21	0	2.52	0.00168	0	0.6	9.00E-05	0	0.99	0.00018
GO:0019199	transmembrane receptor protein kinase ac...	584	64	70.02	0.00181	10	16.57	7.70E-05	17	27.65	0.00018
GO:0004591	oxoglutarate dehydrogenase (succinyl-tra...	12	4	1.44	0.00124	0	0.34	0.00032	0	0.57	0.00045
GO:0016491	oxidoreductase activity	5531	934	663.16	0.00145	268	156.89	0.00309	363	261.84	0.00056

GO:0043559	insulin binding	11	1	1.32	0.01426	0	0.31	0.00362	1	0.52	0.00061
GO:0035613	RNA stem-loop binding	14	0	1.68	0.0019	0	0.4	0.00049	0	0.66	0.00071
GO:0008094	DNA-dependent ATPase activity	253	14	30.33	0.00332	5	7.18	0.00027	6	11.98	0.00074
GO:0002161	aminoacyl-tRNA editing activity	55	3	6.59	0.01324	0	1.56	0.00114	2	2.6	0.00084
GO:0008137	NADH dehydrogenase (ubiquinone) activity	111	11	13.31	0.0033	0	3.15	0.0004	2	5.25	0.00085
GO:0016301	kinase activity	6813	792	816.87	0.0068	160	193.26	0.00226	256	322.54	0.00085
GO:0016504	peptidase activator activity	58	7	6.95	0.01781	0	1.65	0.00035	1	2.75	0.00103
GO:0031406	carboxylic acid binding	537	93	64.39	0.0362	25	15.23	0.00041	35	25.42	0.00499
GO:0004843	ubiquitin-specific protease activity	56	3	6.71	0.00511	2	1.59	0.00064	5	2.65	0.00112
GO:0004318	enoyl-[acyl-carrier-protein] reductase (...	20	1	2.4	0.00273	0	0.57	0.00096	0	0.95	0.00128

Table S4 Characterization of annotated Go term (Cellular component) Unigenes induced by stripe rust pathogen and powdery mildew pathogen.

GO.ID	Term	Annotated unigenes	*Bgt* stress only DEGs			overlapped DEGs			*Pst* stress only DEGs		
			Significant	Expected	KS	Significant	Expected	KS	Significant	Expected	KS
GO:0009536	plastid	14615	2117	1865.41	2.50E-13	648	453.69	0.01134	1022	755.09	2.50E-13
GO:0016021	integral to membrane	5054	814	645.08	5.60E-13	212	156.89	1.30E-05	314	261.12	1.90E-05
GO:0009535	chloroplast thylakoid membrane	911	212	116.28	4.20E-10	98	28.28	1.20E-05	130	47.07	1.40E-07
GO:0016023	cytoplasmic membrane-bounded vesicle	9940	1418	1268.71	1.10E-09	324	308.56	1.10E-09	486	513.56	1.90E-08
GO:0044428	nuclear part	8973	1311	1145.28	3.40E-09	305	278.54	0.00087	567	463.6	7.10E-05
GO:0044446	intracellular organelle part	19487	2897	2487.26	3.70E-09	742	604.93	1.80E-05	1283	1006.81	5.80E-07
GO:0005759	mitochondrial matrix	618	82	78.88	6.10E-09	22	19.18	1.00E-09	35	31.93	8.70E-09
GO:0070013	intracellular organelle lumen	8210	1169	1047.9	6.60E-09	258	254.86	0.00539	471	424.18	0.09895
GO:0009941	chloroplast envelope	2049	379	261.53	6.60E-07	158	63.61	1.00E-04	235	105.86	1.30E-06
GO:0005789	endoplasmic reticulum membrane	1046	121	133.51	9.20E-07	26	32.47	1.80E-06	42	54.04	1.80E-07
GO:0043332	mating projection tip	25	1	3.19	1.50E-06	0	0.78	8.10E-08	0	1.29	1.70E-07
GO:0005793	endoplasmic reticulum-Golgi intermediate...	111	12	14.17	1.70E-06	2	3.45	2.80E-08	3	5.73	4.90E-08
GO:0005576	extracellular region	6026	945	769.14	2.30E-06	269	187.06	0.04149	379	311.34	0.0767
GO:0000131	incipient cellular bud site	27	1	3.45	3.20E-06	0	0.84	1.70E-07	0	1.39	3.70E-07
GO:0009507	chloroplast	9159	1527	1169.02	5.10E-06	510	284.32	0.0627	782	473.21	0.0057
GO:0005935	cellular bud neck	78	4	9.96	6.80E-06	0	2.42	7.10E-06	1	4.03	1.70E-07
GO:0000329	fungal-type vacuole membrane	60	1	7.66	8.10E-06	0	1.86	4.40E-07	0	3.1	1.30E-06
GO:0005628	prospore membrane	24	0	3.06	2.00E-05	0	0.75	1.50E-06	0	1.24	3.00E-06
GO:0005743	mitochondrial inner membrane	905	127	115.51	3.80E-05	31	28.09	6.50E-08	50	46.76	7.40E-07
GO:0005783	endoplasmic reticulum	3393	460	433.07	0.0001	93	105.33	0.0104	144	175.3	0.0566
GO:0010287	plastoglobule	266	67	33.95	0.00011	37	8.26	0.00127	46	13.74	0.00024
GO:0031981	nuclear lumen	7003	943	893.84	0.00016	205	217.39	0.02227	397	361.82	0.00027
GO:0005835	fatty acid synthase complex	28	0	3.57	0.00018	0	0.87	3.50E-05	0	1.45	5.40E-05

GO:0005886	plasma membrane	12757	1830	1628.26	0.00018	405	396.01	0.51917	670	659.1	0.52861
GO:0009705	plant-type vacuole membrane	520	113	66.37	0.0002	37	16.14	0.18382	52	26.87	0.08504
GO:0031298	replication fork protection complex	8	0	1.02	0.00028	0	0.25	5.00E-05	0	0.41	7.70E-05
GO:0005773	vacuole	5028	803	641.76	0.0004	217	156.08	0.14006	318	259.78	0.15262
GO:0030479	actin cortical patch	27	1	3.45	0.00041	0	0.84	4.00E-05	0	1.39	7.40E-05
GO:0044425	membrane part	8459	1266	1079.68	0.00044	301	262.59	0.00705	461	437.04	0.03737
GO:0005934	cellular bud tip	25	1	3.19	0.00045	0	0.78	9.80E-05	0	1.29	0.00015
GO:0005615	extracellular space	481	58	61.39	0.00046	9	14.93	1.10E-06	14	24.85	1.10E-05
GO:0009570	chloroplast stroma	2232	400	284.89	0.00048	173	69.29	0.00168	260	115.32	7.80E-07
GO:0030126	COPI vesicle coat	107	22	13.66	0.00052	2	3.32	0.00024	2	5.53	0.00052
GO:0000775	chromosome, centromeric region	227	17	28.97	0.00058	3	7.05	0.00077	5	11.73	8.80E-05
GO:0000178	exosome (RNase complex)	35	5	4.47	0.00061	1	1.09	0.00036	1	1.81	0.00058
GO:0005782	peroxisomal matrix	123	18	15.7	0.00075	4	3.82	0.00349	8	6.35	0.00245
GO:0022625	cytosolic large ribosomal subunit	634	56	80.92	0.14578	2	19.68	0.00031	24	32.76	1.00E-05
GO:0022627	cytosolic small ribosomal subunit	396	46	50.54	0.01224	2	12.29	9.60E-05	8	20.46	0.00018
GO:0033588	Elongator holoenzyme complex	10	1	1.28	0.00644	0	0.31	0.00202	1	0.52	0.00025
GO:0042645	mitochondrial nucleoid	98	12	12.51	0.02617	5	3.04	0.00084	8	5.06	0.00036
GO:0005811	lipid particle	364	33	46.46	0.00711	1	11.3	7.80E-05	4	18.81	0.00046
GO:0031965	nuclear membrane	168	15	21.44	0.00449	1	5.22	0.0006	4	8.68	0.00049
GO:0019028	viral capsid	22	5	2.81	0.00182	0	0.68	0.00029	0	1.14	0.00056
GO:0030139	endocytic vesicle	271	26	34.59	0.04135	2	8.41	0.00528	12	14	0.0007
GO:0045252	oxoglutarate dehydrogenase complex	22	5	2.81	0.00101	1	0.68	0.00041	1	1.14	0.00077
GO:0005779	integral to peroxisomal membrane	26	2	3.32	0.01676	1	0.81	0.00618	3	1.34	0.00081
GO:0048471	perinuclear region of cytoplasm	672	54	85.77	0.17585	12	20.86	0.00016	20	34.72	0.001
GO:0044444	cytoplasmic part	38619	5000	4929.2	0.01123	1273	1198.83	1.60E-05	2043	1995.28	0.99609
GO:0045254	pyruvate dehydrogenase complex	32	8	4.08	0.00171	1	0.99	0.0006	2	1.65	0.00112

**GO:0009277**	**fungal-type cell wall**	193	25	24.63	0.00251	0	5.99	0.00073	0	9.97	0.00205
**GO:0030136**	**clathrin-coated vesicle**	348	50	44.42	0.05338	7	10.8	0.00075	12	17.98	0.002
**GO:0030127**	**COPII vesicle coat**	28	3	3.57	0.00459	0	0.87	0.00076	0	1.45	0.00128
**GO:0030496**	**midbody**	231	19	29.48	0.03794	7	7.17	0.00082	11	11.93	0.00134
**GO:0030134**	**ER to Golgi transport vesicle**	68	10	8.68	0.00455	0	2.11	0.00087	0	3.51	0.00137
**GO:0030445**	**yeast-form cell wall**	85	12	10.85	0.00196	0	2.64	0.00097	0	4.39	0.00179

Table S5 Significant KEGG pathway of Stimulus-Specific Responses to *Pst* versus *Bgt* directly.

KEGG pathway	Correct-p value		
	1 dpi	2 dpi	3 dpi
ko00360 Phenylalanine metabolism	2.02E-09	1(0.78)	1
ko03010 Ribosome	3.59E-08	1(2.43)	1(1.98)
ko00940 Phenylpropanoid biosynthesis	2.38E-07	1(0.84)	0.5222380
ko00400 Phenylalanine, tyrosine and tryptophan biosynthesis	0.0005789	1(0.84)	1(2.58)
ko00945 Stilbenoid, diarylheptanoid and gingerol biosynthesis	0.0119933	1(0.7)	0.5003830
ko00910 Nitrogen metabolism	0.0336965	1	0.2145717
ko00941 Flavonoid biosynthesis	0.2339049	1	0.0712646
ko00904 Diterpenoid biosynthesis	0.2650820	0.0002160	1
ko00592 alpha-Linolenic acid metabolism	1(0.57)	0.0021489	0.0012211
ko00280 Valine, leucine and isoleucine degradation	1(0.64)	0.0028822	0.3054773
ko00402 Benzoxazinoid biosynthesis	–	0.0036764	0.0236634
ko00196 Photosynthesis - antenna proteins	1(4.61)	0.0136544	1
ko00906 Carotenoid biosynthesis	–	0.0467859	0.0494775
ko00195 Photosynthesis	1(3.32)	0.0860785	0.0281121
ko00591 Linoleic acid metabolism	1(0.56)	0.2615313	0.0082961
ko00710 Carbon fixation in photosynthetic organisms	1(1.21)	0.6822167	0.0579076

Table S6. Specific KEGG pathway of DEGs caused by distinct pathogen and corresponding disturbed enzymes

KEGG pathway	Stress	Related enzymes and K number
ko03020 RNA polymerase	Bgt	RPA1(K02999),RPA2(K03002),RPB1(K03006),RPC1(K03018),RPC37(K14721)
ko03030 DNA replication	Bgt	DpoI(K02335), RFA1(K07466), RFA2/4(K10739), RNAseH2A(K10743)
ko03420 Nucleotide excision repair	Bgt	DpoI(K02335), RBX1(K03868), DDB2(K10140), XPC(K10838), XPG(K10846),RPA(K07466,K10739)
ko03430Mismatch repair	Bgt	RPA(RFA1 K07466, RFA2 K10739)
ko03450 Non-homologous end-joining	Bgt	Ku80(K10885), Rad50(K10866) and Mre11(K10865)
ko02010 ABC transporters	Bgt	ABCB1(K05658)
ko00780 Biotin metabolism	Bgt	biotin synthase bioB (K01012)
ko00785 Lipoic acid metabolism	Bgt	lipoyl(octanoyl) transferase lipB (K03801)
ko00510 N-Glycan biosynthesis	Bgt	ALG7 (K01001),dolichyldiphosphatase (K07252),STT(K07151),OST(K12666, K12670),MAN1(K01230),MGAT1(K00726),MGAT2(K00736),MGAT3(K00737)
ko00062 Fatty acid elongation in mitochondria	Bgt	MECR (K07512)
ko00430 Taurine and hypotaurine metabolism	Bgt	GAD(K01580), ggt (K00681), ADO (K10712)
ko00603Glycosphingolipid biosynthesis - globoseries	Pst	galA(K07407)
ko00531Glycosaminoglycan degradation	Pst	HGSNAT(K10532)
ko04140Regulation of autophagy	Pst	ATP1(K08269), VPS15(K08333)
ko00232Caffeine metabolism	Pst	urate oxidase(K00365)

Table S7. The top 1% up and down regulated DEGs at each timepoints.

Unigene #ID	S1	S2	S3	P1	P2	P3	Species	Discription	Biological Process
T4_Unigene_BMK.44411	6*	2	3	4	5	1	Aegilops tauschii	Protein fluG	respiratory burst involved in defense response
T19_Unigene_BMK.65111	1	6*	5	4	2	3	Aegilops tauschii	hypothetical protein F775_28045	SAR, SA mediated signaling pathway
T13_Unigene_BMK.39881	3	6*	5	2	1	4	Triticum urartu	Phytosulfokine receptor 2	regulation of defense response
T19_Unigene_BMK.48391	1	6*	5	4	2	3	Aegilops tauschii	Putative tyrosine-protein phosphatase	negative regulation of MAPkinase activity
T16_Unigene_BMK.19639	4	6*	5	1	2	3	Aegilops tauschii	Lectin-domain containing receptor kinase A4.2	transmembrane receptor protein Ser/Thr signaling
T10_Unigene_BMK.8012	5	4	6	1*	2*	3	Brachypodium distachyon	peptide transporter PTR2-like	defense response to bacterium
T10_Unigene_BMK.1224	5	4	6	1*	2	3	Arabidopsis thaliana	L-type lectin-domain containing receptor kinase IX.1	regulation of innate immune response
T10_Unigene_BMK.9286	5	4	3	1*	2	6	Triticum aestivum	hemoglobin 1	SAR, SA mediated signaling pathway
T10_Unigene_BMK.68288	5	6	4	1*	2	3	Oryza sativa	Chitinase 2	innate immune response
T7_Unigene_BMK.50460	3	2	1	6*	5*	4	Triticum urartu	Poly(ADP-ribose) glycohydrolase 1	defense response to fungus
T13_Unigene_BMK.29121	5	6	4	1*	2	3	Hordeum vulgare	WRKY transcription factor 18, partial	defense response to fungus
T10_Unigene_BMK.65192	2	4	5	1*	6	3	Pyrus pyrifolia	ACC oxidase	defense response
T19_Unigene_BMK.57259	2	3	1	6	5*	4	Aegilops tauschii	Tyrosine N-monooxygenase	defense response by callose deposition in cell wall
T13_Unigene_BMK.38342	4	5	6	2	1*	3*	Arabidopsis thaliana	Alpha-dioxygenase 1	systemic acquired resistance
T19_Unigene_BMK.60205	3	1	2	4	5	6*	Oryza sativa	Probable 4-coumarate--CoA ligase 2	defense response to fungus
T19_Unigene_BMK.64156	3	4	1	2	5	6*	Oryza sativa	12-oxophytodienoate reductase 1	defense response
T1_Unigene_BMK.14166	1*	2	3	6	4	5	Puccinia graminis	hypothetical protein PGTG_15745	defense response
T7_Unigene_BMK.17207	4	3*	2*	1	5	6	Aegilops tauschii	Putative disease resistance RPP13-like protein 3	aerobic electron transport chain
T19_Unigene_BMK.51512	3*	2	1	5	6*	4*	Nicotiana tabacum	Benzyl alcohol O-benzoyltransferase	phenylpropanoid biosynthetic process
T10_Unigene_BMK.74375	3	6	5	1*	2	4	Hordeum vulgare	Agmatine coumaroyltransferase-2	phenylpropanoid biosynthetic process
T4_Unigene_BMK.35589	4	1	2	3	5	6*	Arabidopsis thaliana	Cytochrome P450 71B16	lignin biosynthetic process
T19_Unigene_BMK.28596	3	2	1	4	5	6*	Aegilops tauschii	Anthranilate N-benzoyltransferase protein 1	lignin biosynthetic process
T10_Unigene_BMK.33608	6*	2	1	3	5*	4*	Ricinus communis	Probable terpene synthase 13	sesquiterpene biosynthetic process
T19_Unigene_BMK.66244	4	2	1	3	6*	5*	Aegilops tauschii	Lipoxygenase 2.2, chloroplastic	jasmonic acid biosynthetic process
T10_Unigene_BMK.7412	4	5	6	1*	2	3	Aegilops tauschii	Aromatic-L-amino-acid decarboxylase	dopamine biosynthetic process
T16_Unigene_BMK.3617	5	6	4	1*	2	3	Triticum urartu	Aromatic-L-amino-acid decarboxylase	dopamine biosynthetic process
T10_Unigene_BMK.2017	3	6	4	1*	2	5	Hordeum vulgare	tryptophan decarboxylase	dopamine biosynthetic process

T19_Unigene_BMK.65657	3	2	1	6	5*	4*	Arabidopsis thaliana	UDP-glycosyltransferase 75D1	flavonoid biosynthetic process
T16_Unigene_BMK.66660	5	3	1	2	6*	4	Brachypodium distachyon	UDP-glycosyltransferase 73C1-like	flavonol biosynthetic process
T7_Unigene_BMK.55212	4*	2	1	6	3	5*	Aegilops tauschii	Linalool synthase, chloroplastic	isoprenoid biosynthetic process
T19_Unigene_BMK.32541	5	2	1	3	4	6*	Swertia mussotii	Geraniol 8-hydroxylase	diterpenoid biosynthetic process
T4_Unigene_BMK.31253	3	1	2	4	6*	5*	Mus musculus	Serine/arginine repetitive matrix protein 2	cellular macromolecule biosynthetic process
T19_Unigene_BMK.63278	4	1	2	3	6*	5*	Arabidopsis thaliana	Probable isoaspartyl peptidase/L-asparaginase 2	autophagic cell death
T10_Unigene_BMK.40836	6*	1	3	2	5	4	Hordeum vulgare	predicted protein	oxidation-reduction process
T19_Unigene_BMK.36612	6*	2	1	3	5	4	Aegilops tauschii	putative Cytochrome P450 71D11	oxidation-reduction process
T4_Unigene_BMK.36102	6*	1	2	4	5	3	Aegilops tauschii	hypothetical protein F775_52192	oxidation-reduction process
T7_Unigene_BMK.35938	3	2	1*	4	6	5	Brassica rapa subsp. chinensis	ribulose 1,5-bisphosphate carboxylase/oxygenase	oxidation-reduction process
T10_Unigene_BMK.79471	4	6	5	1*	2*	3	Aegilops tauschii	Putative flavin-containing monooxygenase1	oxidation-reduction process
T10_Unigene_BMK.39391	2	6	3	1*	4	5	Oryza sativa	Laccase-19	oxidation-reduction process
T10_Unigene_BMK.2939	4	5	6	1*	4*	3	Triticum aestivum	class III peroxidase	oxidation-reduction process
T10_Unigene_BMK.78990	4	5	6	1*	2	3	Oryza sativa	Ent-isokaurene C2-hydroxylase	oxidation-reduction process
T19_Unigene_BMK.38731	1	5	3*	2	6	4	Aegilops tauschii	Cytochrome P450 71D7	oxidation-reduction process
T10_Unigene_BMK.9714	3	5	6	1*	2	4	Triticum urartu	Cytochrome P450 71D18	oxidation-reduction process
T10_Unigene_BMK.77447	3	5	6	1*	2	4	Triticum urartu	Cytochrome P450 99A2	oxidation-reduction process
T13_Unigene_BMK.10824	4	5	6	1*	2*	3	Triticum urartu	Peroxidase	oxidation-reduction process
T10_Unigene_BMK.64805	4	5	6	2	1*	3*	Glarea lozoyensis	putative fumarate reductase	oxidation-reduction process
T19_Unigene_BMK.50234	4	1	2	3	6*	5	Aegilops tauschii	Nitrate reductase (NAD(P)H)	oxidation-reduction process
T16_Unigene_BMK.30983	4	5	6	2	1*	3*	Mycobacterium tuberculosis	Putative monooxygenase Rv1533	oxidation-reduction process
T13_Unigene_BMK.85907	4	5	6	3	1*	2	Blumeria graminis	catalase/peroxidase	oxidation-reduction process
T19_Unigene_BMK.39009	3	6*	2	1	4	5*	Triticum urartu	Hyoscyamine 6-dioxygenase	oxidation-reduction process
T10_Unigene_BMK.73994	4	5	6	1*	2	3	Triticum urartu	3β-hydroxysteroid-dehydrogenase/decarboxylase isoform 1	oxidation-reduction process
T4_Unigene_BMK.49818	1*	2	3	5	4	6	Pgt	hypothetical protein PGTG_06207	oxidation-reduction process
T10_Unigene_BMK.8063	2	4	5	1*	6	3	Aspv	RNA replication protein	oxidation-reduction process
T10_Unigene_BMK.4777	2	4	5	1*	6	3	Apple stem pitting virus	RNA replication protein	oxidation-reduction process
T10_Unigene_BMK.3559	2	4	5	1*	6	3	Apple stem pitting virus	RNA replication protein	oxidation-reduction process;mRNA methylation
T13_Unigene_BMK.46461	1	5*	6	4	2	3	Hordeum vulgare	predicted protein	tricarboxylic acid cycle
T13_Unigene_BMK.34608	1	4	5	6*	2	3	Triticum aestivum	isocitrate lyase	tricarboxylic acid cycle
T13_Unigene_BMK.46459	1	4	6*	5	2	3	Zea mays	Malate synthase, glyoxysomal	tricarboxylic acid cycle

T13_Unigene_BMK.34608	1	4	5*	6*	2	3	Triticum aestivum	isocitrate lyase	tricarboxylic acid cycle
T19_Unigene_BMK.64560	6*	2	1	3	4	5*	Aegilops tauschii	Putative UDP-glucosyltransferase	response to karrikin
T16_Unigene_BMK.16938	2	5	6*	3	1	4	Arabidopsis thaliana	Probable galactinol-sucrose galactosyltransferase6	response to karrikin
T19_Unigene_BMK.52305	3	1	2	6*	2	5*	Homo sapiens	Serine/arginine repetitive matrix protein 1	response to karrikin
T10_Unigene_BMK.42975	4	5	6	1*	2	3	Aegilops tauschii	Luminal-binding protein	cellular response to antibiotic
T13_Unigene_BMK.49119	4	5	6	1*	2	3*	Botryotinia fuckeliana	elongation factor 1-alpha	cellular response to drug
T16_Unigene_BMK.86937	3*	2	1	4	5*	6*	Triticum aestivum	chalcone synthase	response to jasmonic acid stimulus
T1_Unigene_BMK.41264	1	2	3	5	4	6*	Triticum aestivum	MYB-related protein	response to hormone stimulus
T19_Unigene_BMK.62625	3	1	2	4	6*	5*	Oryza sativa	Putative calmodulin-like protein 2	hyperosmotic salinity response
T10_Unigene_BMK.9898	1	6*	4	3	2	5	Triticum urartu	Cold shock protein CS66	response to stimulus
T13_Unigene_BMK.44172	1	6*	5	3	2	4	Arabidopsis thaliana	BTB/POZ and TAZ domain-containing protein 2	response to stimulus
T10_Unigene_BMK.10044	4	5	6	2	1*	3	Marssonina brunnea f. sp. &apos	chalcone and stilbene synthase domain-containing protein	response to stimulus
T19_Unigene_BMK.48456	3	2	1	4	6	5*	Arabidopsis thaliana	UDP-glycosyltransferase 90A2	response to stimulus
T10_Unigene_BMK.68445	4	5	6	2	1*	3	Sclerotinia sclerotiorum	enolase	filamentous growth of apop…to biotic stimulus
T16_Unigene_BMK.30394	5	6	4	2*	1*	3*	Sclerotinia sclerotiorum	hypothetical protein SS1G_03130	Signal transduction mechanisms
T10_Unigene_BMK.72147	3	6	4	1*	2	5	Arabidopsis thaliana	Benzoate--CoA ligase, peroxisomal	GA mediated signaling pathway
T10_Unigene_BMK.62784	4	5	6	3	1*	2	Eutypa lata	putative replicase readthrough protein	binding (GO:0005488)
T1_Unigene_BMK.22881	1*	2	3	5	6	4	Pgt	hypothetical protein PGTG_04611	protein binding
T4_Unigene_BMK.24386	1*	2	3	5	4	6	Pgt	hypothetical protein PGTG_04693	biological regulation
T1_Unigene_BMK.39163	1	2	3	6	4	5*	Triticum aestivum	MYB-related protein	regulation of protein homodimerization activity
T10_Unigene_BMK.4959	3	5	4	1*	2	6	Arabidopsis thaliana	Putative transcription factor bHLH041	regulation of transcription
T4_Unigene_BMK.16776	1*	2	3	5	6	4	Medicago truncatula	hypothetical protein MTR_7g109740	regulation of cellular process
T19_Unigene_BMK.62039	5*	3*	1	2	4*	6*	Aegilops tauschii	hypothetical protein F775_20874	cellular process
T16_Unigene_BMK.82348	4	6	5	1*	2	3	Hordeum vulgare	predicted protein	RNA processing
T19_Unigene_BMK.58369	4	1	2	3	5	6*	Arabidopsis thaliana	F-box only protein 13	RNA metabolic process
T16_Unigene_BMK.71879	4	5	6	3	1*	2*	Eutypa lata	putative replicase readthrough protein	metabolic process
T19_Unigene_BMK.61704	4	2	1	3	6	5*	Triticum urartu	(E)-beta-farnesene synthase	metabolic process
T4_Unigene_BMK.1295	2	3	1*	4	6	5	Brachypodium distachyon	PREDICTED: UDP-glycosyltransferase 89B1-like	metabolic process
T7_Unigene_BMK.49453	5	1	2	3	6*	4	Castanea crenata	Agglutinin	protein catabolic process
T19_Unigene_BMK.22212	4	1	2	5	3	6*	Synechococcus	ATP-dependent Clp protease adapter protein	protein catabolic process
T19_Unigene_BMK.60696	4*	2	1	5	6*	3*	Clarkia breweri	Benzyl alcohol O-benzoyltransferase	macromolecule metabolic process

T13_Unigene_BMK.22137	4	5	6	3	1*	2*	Eutypa lata	putative replicase readthrough protein	macromolecule metabolic process
T19_Unigene_BMK.55835	4	1	2	3	6	5*	Clarkia breweri	Salicylate O-methyltransferase	lipid metabolic process
T10_Unigene_BMK.42111	3	4	2*	1	5	6	Blastocladiella emersonii	apocytochrome b	electron transport chain
T10_Unigene_BMK.37883	4	2*	3*	1	5	6	Lingulodinium polyedrum	cytochrome c oxidase subunit I	electron transport chain
T4_Unigene_BMK.35276	3	1	2	4	5*	6*	Triticum urartu	Bidirectional sugar transporter SWEET2b	carbohydrate transport
T10_Unigene_BMK.2206	2	4	5	1*	6	3	Aspv	Capsid protein	transport
T19_Unigene_BMK.68629	4*	2	1	3	5*	6*	Triticum aestivum	boron transporter 2	borate transmembrane transport
T10_Unigene_BMK.63925	4	5	6	1*	2	3	Blumeria graminis	plasma membrane H+-ATPase	mycelium development
T19_Unigene_BMK.52800	2	6	5*	4	1	3	Aegilops tauschii	hypothetical protein F775_28626	developmental process
T19_Unigene_BMK.63651	6*	2	3	5	1	4	Brachypodium distachyon	pyruvate kinase isozyme G, chloroplastic-like	phosphorylation
T7_Unigene_BMK.36614	6*	2	1	5	4	3	Aegilops tauschii	Profilin	actin cytoskeleton organization
T13_Unigene_BMK.45944	4	5	6	1*	2	3	Aegilops tauschii	delta-cadinene synthase isozyme XC14	terpene synthase activity
T19_Unigene_BMK.67352	2	3	1	4	5	6*	Petunia hybrida	Flavonoid 3'-monooxygenase	monooxygenase activity
T19_Unigene_BMK.54041	3*	2*	1	4	5*	6*	Hordeum vulgare	Low molecular mass early light-inducible protein HV90	chloroplast membrane
T13_Unigene_BMK.67646	2	6*	1	4	3*	5	Hordeum vulgare	Low molecular mass early light-inducible protein HV90	chloroplast membrane
T16_Unigene_BMK.90713	2	6	5*	4	1	3	Arabidopsis thaliana	Lysine-rich arabinogalactan protein 19	unknown
T10_Unigene_BMK.70553	2	5	6*	4	1	3	Aegilops tauschii	Bowman-Birk type trypsin inhibitor TI1	unknown
T10_Unigene_BMK.12670	3	2	1	4	5*	6	Hordeum vulgare	High molecular mass early light-inducible protein HV58	unknown
T4_Unigene_BMK.35879	2	3	1	4	5*	6*	Hordeum vulgare	High molecular mass early light-inducible protein HV58	unknown
T1_Unigene_BMK.40864	1*	2	3	4	6	5	Pst	differentiation-related protein 1	unknown
T1_Unigene_BMK.22888	1*	2	3	5	4	6	Pgt	hypothetical protein PGTG_14956	unknown
T1_Unigene_BMK.18134	1*	2	3	6	4	5	Pgt	hypothetical protein PGTG_06171	unknown
T4_Unigene_BMK.21663	1*	2	3	4	6	5	Aegilops tauschii	hypothetical protein F775_23939	unknown
T7_Unigene_BMK.57960	1	6*	2	4	5	3	Triticum urartu	hypothetical protein TRIUR3_11313	unknown
T16_Unigene_BMK.29897	3	5	6*	4	1	2	Triticum urartu	hypothetical protein TRIUR3_08548	unknown
T10_Unigene_BMK.69134	3	6	5	1*	2	4	Aegilops tauschii	hypothetical protein F775_31186	unknown
T16_Unigene_BMK.92477	5	4	6	3	1*	2	Aegilops tauschii	hypothetical protein F775_27997	unknown
T4_Unigene_BMK.30077	4	2	1	3	6*	5	Triticum urartu	hypothetical protein TRIUR3_29839	unknown
T13_Unigene_BMK.71039	4	5	6	3	1*	2*	Exophiala dermatitidis	hypothetical protein HMPREF1120_11008	unknown
T1_Unigene_BMK.7762	3	6	5	4	1*	2*	Aegilops tauschii	hypothetical protein F775_26893	unknown
T16_Unigene_BMK.67379	4	5	6	3	1*	2	Triticum aestivum	hypothetical protein	unknown

T4_Unigene_BMK.40145	4	2	1	3	6*	5	Hordeum vulgare	predicted protein	unknown
T4_Unigene_BMK.27233	1	3	2	4	6*	5*	Brachypodium distachyon	uncharacterized protein LOC100821901	unknown
T4_Unigene_BMK.11511	4	1	2	6	3	5*	Brachypodium distachyon	uncharacterized protein LOC100841361	unknown
T1_Unigene_BMK.25312	1*	2	3	5	6	4	-	NS	unknown
T4_Unigene_BMK.360	1*	2	3	4	6	5	-	NS	unknown
T4_Unigene_BMK.2196	1*	2	3	6	5	4	-	NS	unknown
T16_Unigene_BMK.9085	4	5	6	3*	1*	2*	-	NS	unknown
T16_Unigene_BMK.30030	4	5	6	2*	1*	3*	-	NS	unknown
T16_Unigene_BMK.30281	4	5	6	3*	1*	2*	-	NS	unknown
T13_Unigene_BMK.81987	4	5	6	3*	1*	2*	-	NS	unknown
T19_Unigene_BMK.48960	6*	1	2	3	5*	4*	-	NS	unknown
T10_Unigene_BMK.9590	4	5	6	2	1*	3*	-	NS	unknown
T19_Unigene_BMK.63301	1	2	3	5	4*	6	-	NS	unknown
T16_Unigene_BMK.30338	4	5	6	3	1	2*	-	NS	unknown

Note: S1, S2 and S3 mean N9134 infected with stripe rust pathogen CYR 31 at 1, 2 and 3 dpi, respectively; P1, P2 and P3 represent N9134 infected with powdery mildew pathogen E09 at 1, 2 and 3 dpi respectively. The gene expression level was ranked from high to low at all treatments, and marked from 1 to 6. Pink cell indicated the unigene was up regulated at relative treatment comparing with non inoculation control, while grey cell means down regulation conversely.

Table S8 PCR primers used for Q-PCR amplification with cDNAs and PCR with gDNA

Primer	Forward primer sequence	Reverse primer sequence
**T4.38637**	5-TGGGAGACGTTCGACCTGCTGT-3	5-ATCGGCGAGGTGGTGTTGTTGT-3
**T10.2939**	5-GATCATGGCGGTCGTGAAGG-3	5-GCACTCGGACCAGGTGCTGT-3
**T13.48786**	5-TGCAGGCTTCATTCCAATCT-3	5-TAGAAGCTACGCCGTCACAT-3
**T16.15561**	5-TGAGCGGTACTGCTAGAATGGA-3	5-AGTCGAGCCGGGTGGTTTGT-3
**T16.9085**	5-TGAGACTCGGTTATGACAGTAGGAAG-3	5-ACAGGGTAGAAACCCCGTATTTGC-3
**T19.26915**	5-TCTTCTCGCAGCAGCGTCAC-3	5-GTCGTCCGAGCAGCATTCCA-3
**T7.8064**	5-GATGATGGATCCGTGGAGAT-3	5-AGAGGAGAAAGATGGGAGCA-3
**T16.9260**	5-GATGCAACCCACTTGGTCTT-3	5-ACCTCAACACCTCCATGAGC-3

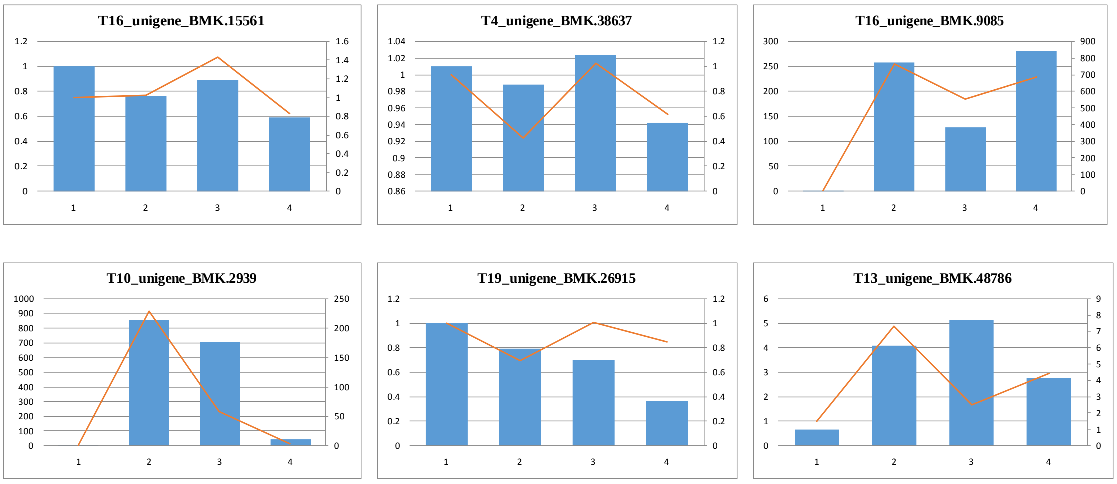

Figure S1. Evaluate the reliability of RNA-Seq with qRT-PCR. Bar graph showed the result of gene expression checked with qRT-PCR, while line graph represent the results of RNA-Seq. The unigenes name was given on the top of each chart.

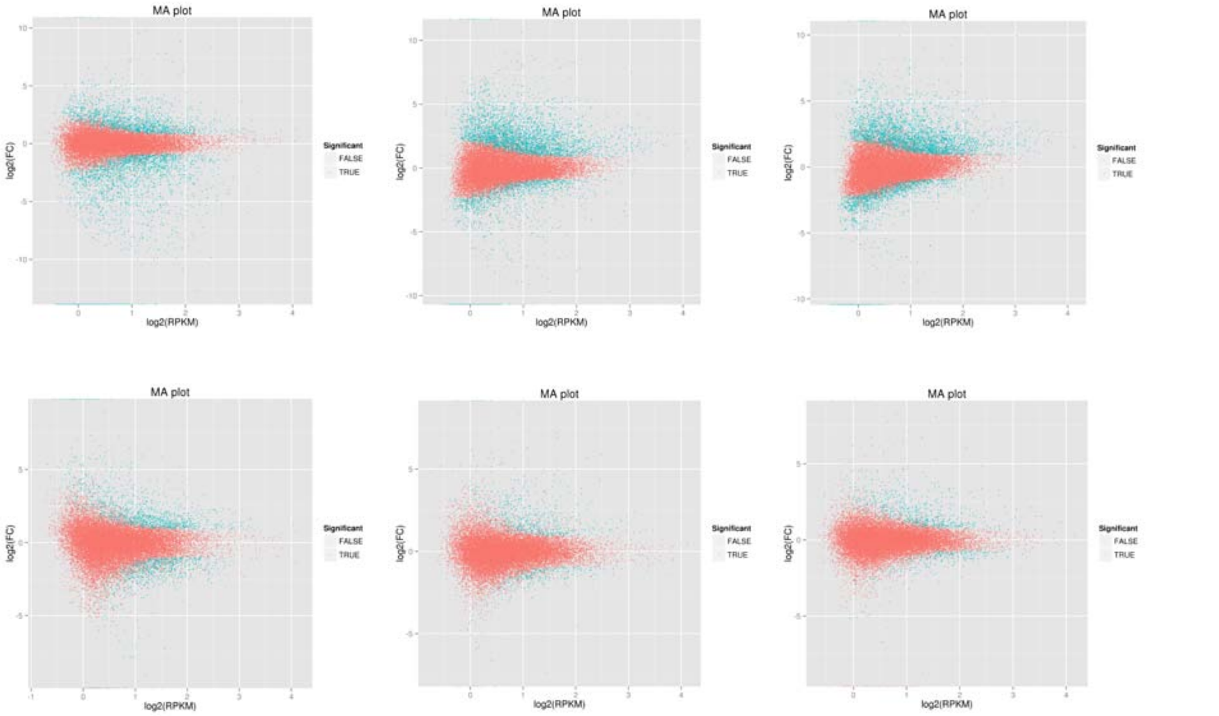

Figure S2. M A Scatter plot analysis of gene expression quantity variability. The effect of different factors and time points on signal variation was analyzed: powdery mildew stress (top panel) and stripe rust stress (bottom panel) at 1 dpi (left), 2 dpi (middle) and 3 dpi (right) respectively. Green signals represent differentially expressed genes and Red signals are false DEGs. X axle is the value of log2 (RPKM) and represents gene expression level. Y axle is the value of log2(FC) and measure variant of expression quantity among test samples.

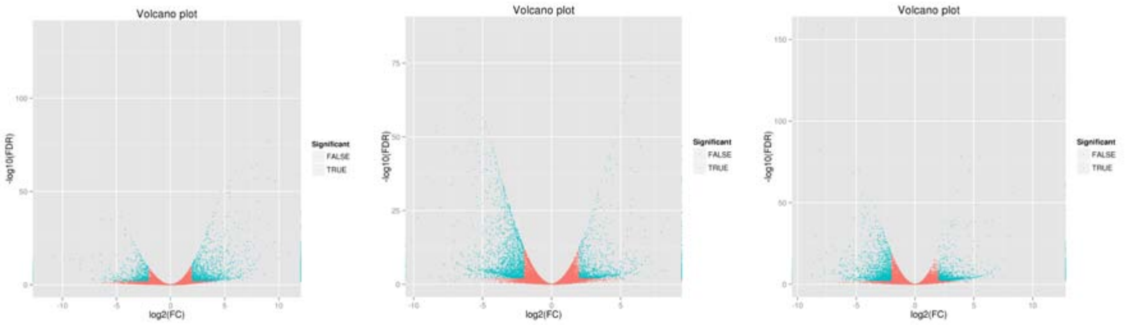

Figure S3. Volcano scatter plot analysis of differentially expression gene. The effect of different time points on signal variation was analyzed: stripe rust stress Vs powdery mildew stress at 1 dpi (left), 2 dpi (middle) and 3 dpi (right). Green signals represent differentially expressed genes and Red signals are false DEGs at the level of FC < 4. X axle is the value of log2(FC) measure variant of expression quantity among treatment groups and Y axle is the value of -log10(FDR) and represents the false discovery rate level.

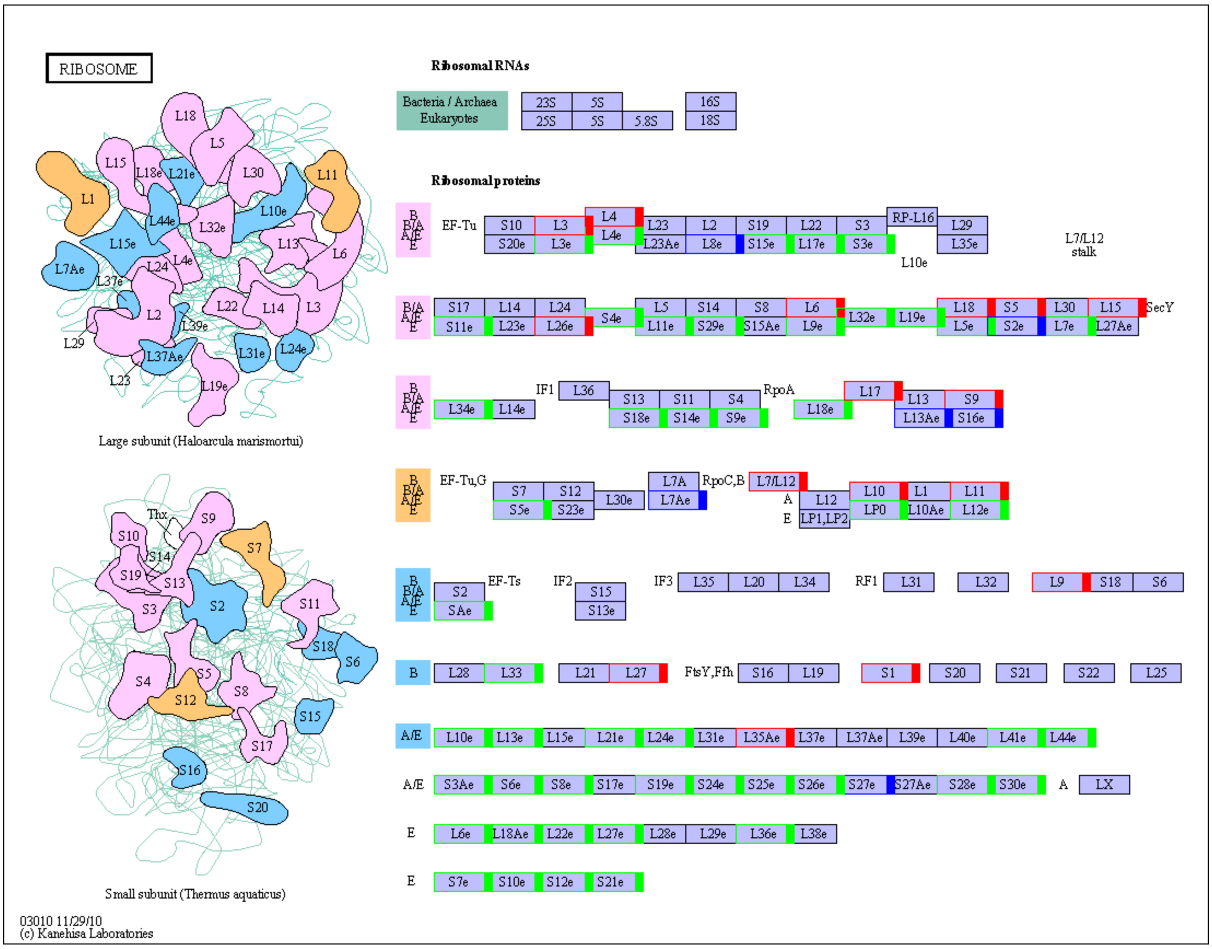

Figure S 4a. Disturbed genes matched with enzymes in ribosome pathway in Bgt infection. Green means that the DEGs encoding corresponding enzyme were up regulated in contrasting with non inoculation; red represent down regulation and blue indicated that DEG unigenes are mixed expressed.

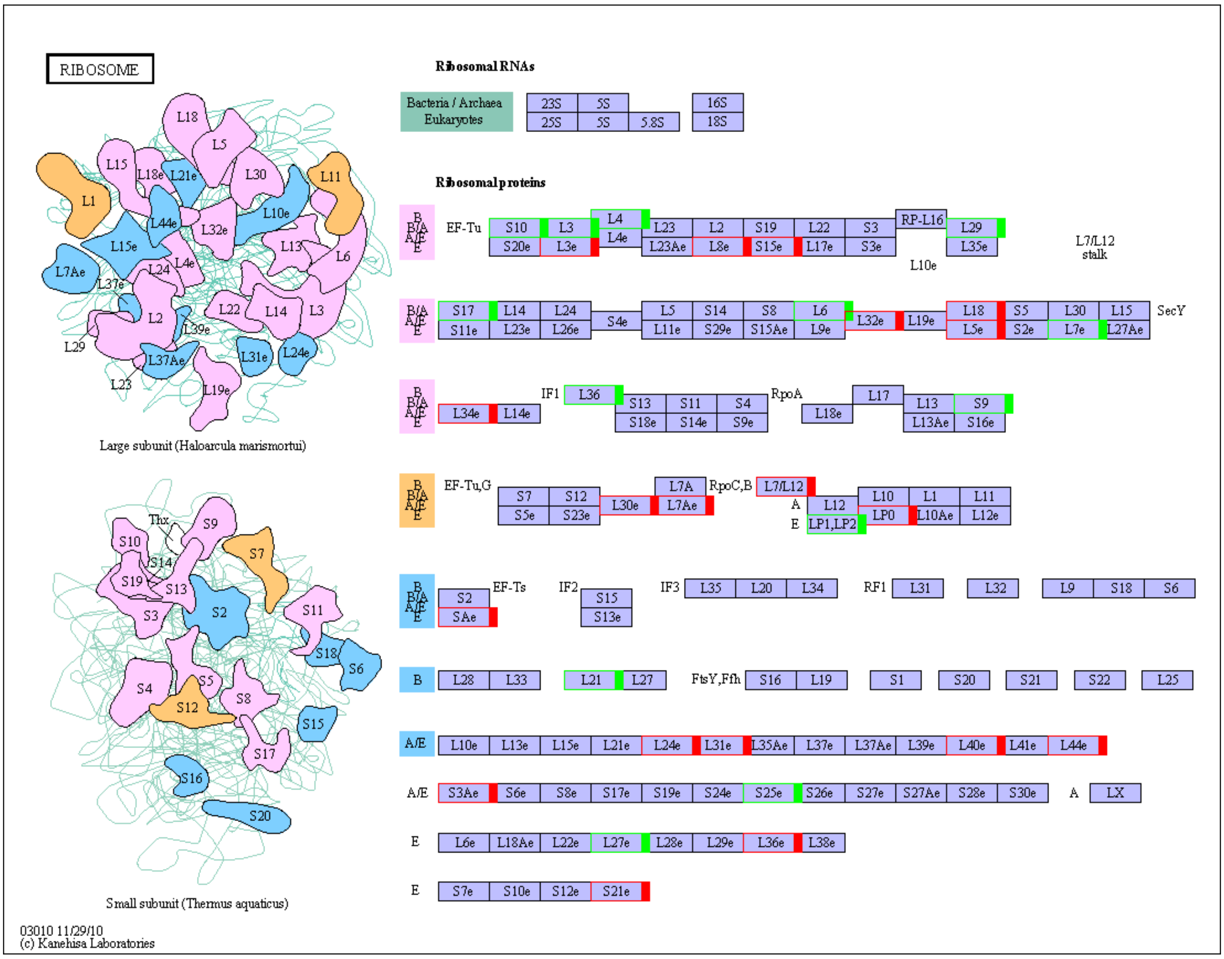

Figure S 4b. Disturbed genes matched with enzymes in ribosome pathway in Pst infection. Green means that the DEGs encoding corresponding enzyme were up regulated in contrasting with non inoculation; red represent down regulation and blue indicated that DEG unigenes are mixed expressed.

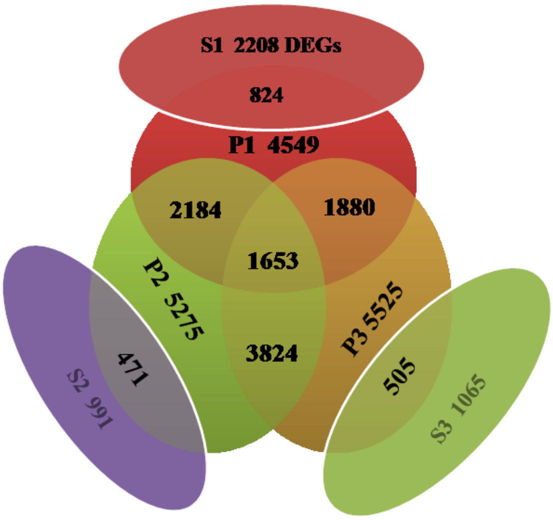

Figure S5 Venn diagram to illustrate the number of differentially expression genes shared by *Pst* and *Bgt* at the same time point, as well as overlapped gene at different time points in *Bgt* stress. Numbers of DE genes in each category are shown. All the lists represented in the treatment groups can be determined from Table 1. S1, S2 and S3: N9134 infected with stripe rust pathogen CYR 31 at 1, 2 and 3 dpi, respectively; P1, P2 and P3: N9134 infected with powdery mildew pathogen E09 at 1, 2 and 3 dpi, respectively.

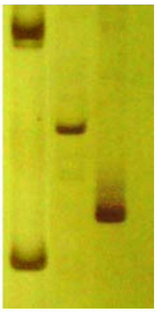

Figure S6. Acrylamide gel electrophoresis (8%) showing the size of the PCR product amplified with gene-specific primers designed from Unigene T16.9260 and T17.8064 (homologous to *Pgt*) in genome DNA of N9134 healthy leaves.
